# Neuronal Network Excitability in Alzheimer’s Disease: The Puzzle of Similar versus Divergent Roles of Amyloid β and Tau

**DOI:** 10.1523/ENEURO.0418-20.2020

**Published:** 2021-04-21

**Authors:** Syed Faraz Kazim, Joon Ho Seo, Riccardo Bianchi, Chloe S. Larson, Abhijeet Sharma, Robert K. S. Wong, Kirill Y. Gorbachev, Ana C. Pereira

**Affiliations:** 1 Department of Neurology and Nash Family Department of Neuroscience, Friedman Brain Institute, Icahn School of Medicine at Mount Sinai, New York, NY 10029; 2 The Robert F. Furchgott Center for Neural and Behavioral Science, and Department of Physiology and Pharmacology, State University of New York (SUNY) Downstate Medical Center, Brooklyn, NY 11203; 3 Ronald M. Loeb Center for Alzheimer’s Disease, Icahn School of Medicine at Mount Sinai, New York, NY 10029

**Keywords:** amyloid β, neuronal excitability, seizures, tau

## Abstract

Alzheimer’s disease (AD) is the most frequent neurodegenerative disorder that commonly causes dementia in the elderly. Recent evidence indicates that network abnormalities, including hypersynchrony, altered oscillatory rhythmic activity, interneuron dysfunction, and synaptic depression, may be key mediators of cognitive decline in AD. In this review, we discuss characteristics of neuronal network excitability in AD, and the role of Aβ and tau in the induction of network hyperexcitability. Many patients harboring genetic mutations that lead to increased Aβ production suffer from seizures and epilepsy before the development of plaques. Similarly, pathologic accumulation of hyperphosphorylated tau has been associated with hyperexcitability in the hippocampus. We present common and divergent roles of tau and Aβ on neuronal hyperexcitability in AD, and hypotheses that could serve as a template for future experiments.

## Significance Statement

Abnormal neuronal network excitability may lead to hypersynchrony, aberrant oscillatory rhythmic activity and interneuron dysfunction, which may contribute to cognitive decline in Alzheimer’s disease (AD). The main goals of this review are the following: (1) to provide an overview of the current knowledge on the association between abnormal network dysfunction and AD; (2) discuss the role of pathologic Aβ and tau on neuronal hyperexcitability; and (3) present potential hypotheses that can be tested for future studies, which could lead to more effective strategies to prevent, diagnose, and manage AD and related disorders.

## Introduction

### Alzheimer’s disease (AD): health care burden and neuropathology

AD is an age-dependent chronic progressive neurodegenerative disorder, and is the leading cause of dementia worldwide (Ballard et al., 2011; Cornutiu, 2015; Alzheimer’s Association, 2019). It is the sixth leading cause of mortality in the United States and affects ∼5.8 million Americans (Alzheimer’s Association, 2019). Worldwide, AD and related dementias affect ∼47 million people (Prince, 2015). In the year 2019, the total health care expenditure for AD and related dementias in United States was nearly 290 billion dollars, making it one of the costliest chronic diseases (Alzheimer’s Association, 2019). To date, there is no effective disease-modifying therapy for AD.

Histopathologically, AD is characterized by two major lesions: amyloid as diffuse and neuritic plaques composed of amyloid β (Aβ) peptide, and neurofibrillary tangles (NFTs) composed of abnormally hyperphosphorylated tau protein (Glenner and Wong, 1984; Grundke-Iqbal et al., 1986a,b). Besides Aβ plaques and NFTs, impairments in adult hippocampal neurogenesis and synaptic plasticity, profound synaptic loss, and neurodegeneration are also major features of AD (Terry et al., 1991; Selkoe, 2002; Scheff and Price, 2003; Scheff et al., 2007; Li et al., 2008; Demars et al., 2010; Shruster et al., 2010; Mu and Gage, 2011). The hippocampal formation in the brain is the hub of learning and memory (Neves et al., 2008; Battaglia et al., 2011), and entorhinal cortex (EC), within the hippocampal formation, is one the first brain regions to be affected by AD pathology (Braak and Braak, 1991, 1995, 1996). Despite the dramatic advances in understanding the molecular pathology underlying neurodegeneration in AD during the past few decades, current knowledge of the physiological basis of memory loss in AD is limited.

### Network dysfunction in AD

Network abnormalities and their contribution to cognitive dysfunction in AD have been reviewed before (Palop and Mucke, 2010a,b, 2016; Zott et al., 2018; Busche and Hyman, 2020; Jun et al., 2020). Network hypersynchrony, altered oscillatory rhythmic activity, interneuron dysfunction, and synaptic depression may be key mediators of cognitive deficits in AD (Palop and Mucke, 2010a,b, 2016). Emerging evidence suggests that brain network alterations begin even decades before the symptomatic onset of AD (Busche and Konnerth, 2016; Nakamura et al., 2017). Besides, recent studies have provided evidence that abnormal neuronal network activities could contribute to the spread of pathology across functionally connected brain circuitry in AD (Wu et al., 2016; Schultz et al., 2018). It can thus be speculated that early brain network dysfunction not only contributes to cognitive dysfunction but also to disease progression in AD.

Brain activity in humans can be evaluated by employing functional magnetic resonance imaging (fMRI), positron emission tomography (PET), single-photon emission computed tomography (SPECT), electroencephalography (EEG), or local field potentials (LFPs) recordings (Palop and Mucke, 2016). In healthy individuals, cognitive tasks increase fMRI signals in particular brain regions (e.g., the hippocampus during learning) but also lead to a profound large-scale deactivation in brain regions that are jointly referred to as the default mode network (DMN; Raichle et al., 2001; Boyatzis et al., 2014; Palop and Mucke, 2016). The DMN includes several brain regions such as the precuneus, posterior cingulate cortex, lateral and inferior parietal cortex, and regions of the temporal and medial prefrontal cortex (Raichle et al., 2001). A consistent fMRI signature of AD, specifically during the early stages of the disease, is hippocampal hyperactivation and reduced deactivation of DMN components during memory encoding tasks (Bookheimer et al., 2000; Dickerson et al., 2005; Celone et al., 2006; Trivedi et al., 2008; Filippini et al., 2009; Sperling et al., 2009; Quiroz et al., 2010; Bakker et al., 2012, 2015; Sepulveda-Falla et al., 2012; Kunz et al., 2015). Early hippocampal hyperactivation was traditionally thought of as a compensatory mechanism for emerging cognitive dysfunction in early AD (Dickerson et al., 2004; Kunz et al., 2015). However, recent evidence points toward a primary pathogenic role of this early hippocampal hyperactivity, and it may play a major role in cognitive decline in AD (Putcha et al., 2011; Bakker et al., 2012, 2015). This will be discussed in detail in later (see below, Seizures and neuronal network hyperexcitability in AD: a late-onset consequence of neurodegeneration or an early component of AD pathophysiology contributing to cognitive impairment? and Beneficial effect of levetiracetam, an antiepileptic drug, on cognition in AD patients and mouse models: evidence for the role of neuronal network hyperexcitability in cognitive impairment?).

Neural oscillations (or brain rhythms) are rhythmic fluctuations of electrical activity in the CNS that emerge because of the physiological properties of different types of neural cells and their interactions (Buzsáki et al., 2012, 2013). Normal neuronal synchrony underlies the generation of oscillatory brain rhythms that promote cognitive functions including memory. Oscillations, from lowest to highest frequencies, are classified into δ, θ, α, β, γ, and sharp-wave ripples (SWRs). γ Oscillations, in particular, are of interest because of their proposed role in organization of functional neural circuits and formation of functional neuronal assemblies, contributing to sensory processing, attention, and memory (Fries, 2015; Lundqvist et al., 2016). Accumulating evidence shows that γ power as well as interareal γ coherence is severely affected in AD patients (Stam et al., 2002; Koenig et al., 2005; Guillon et al., 2017). More recently, Jun and his colleagues demonstrated that remapping capabilities of CA1 cells and grid cells are severely affected by expression of human mutant gene of APP (APP-knockin; APP-KI). The authors found that CA1 neurons from APP-KI mice exhibited reduced firing peaks and less spatial tuning, with lower mean spatial information compared with those of wild-type (WT) CA1 neurons. To investigate the impact of human Aβ precursor protein (hAPP) on remapping of CA1, the authors recorded from CA1 neurons while WT and APP-KI mice were subjected to two different environments. While WT CA1 neurons showed distinct firing patterns with respect to their environment, APP-KI CA1 neurons showed no changes in firing patterns between the environments. Further analyses revealed that fast γ oscillations, but not slow γ oscillations, were found to be diminished in APP-KI mice (Jun et al., 2020). Also, reduced γ is observed in several AD mouse models, remarkably even at the presymptomatic stage (Verret et al., 2012; Iaccarino et al., 2016). Recent studies have demonstrated reduction of disease pathology by induction of γ oscillations via sensory stimulation (γ entrainment using sensory stimulus or GENUS) in AD mouse models (Adaikkan et al., 2019; Martorell et al., 2019). Similarly, alterations in other brain rhythms such as hippocampal SWRs and θ oscillations have also been implicated in AD and will be discussed in detail in later (see High-frequency oscillations or SWRs, epilepsy, and tau; and Phosphorylation of tau reduces hippocampal excitability).

Neural network synchrony and brain oscillatory rhythms are governed by the activity of inhibitory GABAergic interneurons (Buzsáki and Draguhn, 2004). Certain types of inhibitory interneurons such as parvalbumin-positive (PV^+^) or vasoactive intestinal polypeptide-positive (VIP^+^) cells fire mainly during brain states that promote memory encoding (Lapray et al., 2012; Fu et al., 2014). Inhibitory interneuron dysfunction has been linked to network abnormalities in AD (Lapray et al., 2012; Fu et al., 2014; Palop and Mucke, 2016). It was found that impairments of inhibitory interneurons contribute to network hypersynchrony, altered oscillatory rhythms, and behavioral deficits in hAPP-J20 mouse model of AD (Verret et al., 2012). In fact, accumulating evidence implicates inhibitory interneuron dysfunction as a potential common mediator of altered brain rhythms and cognitive impairment in several neuropsychiatric disorders (Sohal et al., 2009; Chao et al., 2010; Marin, 2012; Verret et al., 2012). Recent studies show that modulating the interneuron function ameliorates altered brain rhythms and cognitive impairments in AD and other neurologic disorders (Verret et al., 2012; Hunt et al., 2013; Tong et al., 2014; Dargaei et al., 2018; Martinez-Losa et al., 2018).

In experimental rodent models, high Aβ levels were shown to cause synaptic loss, decrease glutamatergic synaptic transmission and long-term potentiation (LTP), and increase long-term depression (LTD; Hsia et al., 1999; Mucke et al., 2000; Kim et al., 2001; Walsh et al., 2002; Kamenetz et al., 2003; Hsieh et al., 2006; Li et al., 2009; Mucke and Selkoe, 2012). Because of several proposed shared underlying mechanisms, synaptic depression and aberrant excitatory network activity have been hypothesized to be the two faces of the same coin contributing to network dysfunction in AD (Palop and Mucke, 2010a,b). Aβ was reported to block neuronal glutamate uptake at synapses, resulting in glutamate spillover and aberrant activation of extrasynaptic or perisynaptic GluN2B-containing NMDA receptors (NMDARs) and metabotropic glutamate receptors (mGluRs), ultimately leading to enhanced LTD (Hsieh et al., 2006; Li et al., 2009). Aβ-induced NMDAR-dependent and mGluR-dependent LTD can be mimicked by employing the glutamate reuptake inhibitor threo-β-benzyloxyaspartate (TBOA), which can also induce synchronized epileptiform discharges in WT brain slices (Campbell et al., 2014). Thus, Aβ-induced neuronal network hyperexcitability and synaptic depression in AD may be interlinked through common mediator pathways (Palop and Mucke, 2010a,b, 2016).

### Seizures and epilepsy in AD

It has long been known that AD patients are at increased risk of developing seizures and epilepsy (Friedman et al., 2012; Vossel et al., 2013, 2017). Early-onset familial AD (EOFAD), caused by genetic mutations in APP, presenilin 1 (PSEN1), and presenilin 2 (PSEN2), is associated with a remarkable 87-fold higher seizure incidence compared with the general population (Amatniek et al., 2006; Cloyd et al., 2006). In contrast, the late-onset sporadic AD is associated with a 3-fold rise in seizure incidence (Amatniek et al., 2006; Cloyd et al., 2006). Also, AD severity was reported to correlate with seizure occurrence. In prospective studies of mild neurocognitive dysfunction because of probable AD, seizures were found to occur in 0.5–16% of patients (Hauser et al., 1986; Romanelli et al., 1990; Amatniek et al., 2006); however, in more advanced, institutionalized AD patients, the incidence of seizures ranged from 9% to 64% (Sulkava, 1982; Risse et al., 1990; McAreavey et al., 1992).

### Seizures and neuronal network hyperexcitability in AD: a late-onset consequence of neurodegeneration or an early component of AD pathophysiology contributing to cognitive impairment?

A widely prevalent classical notion was that dementia and seizures exemplify two primarily independent disorders, a supportive argument being that not every individual with generalized seizures goes on to develop progressive cognitive dysfunction. Nonetheless, epilepsy does interfere with cognitive development in temporal lobe epilepsy (TLE) individuals with hippocampal sclerosis (Helmstaedter and Elger, 2009). Thus, despite AD being widely known as a risk factor for seizures, seizures in AD were thought to be a consequence of neurodegeneration (Scarmeas et al., 2009). However, during the last decade, several mouse model studies have challenged this notion, and have suggested a different view regarding the relationship between epileptic seizures and AD: instead of being a complication of AD, epileptiform activity including both convulsive and non-convulsive seizures may represent a primary disturbance and contribute to network dysfunction, cognitive impairment, and disease progression in AD (Leonard and McNamara, 2007; Palop et al., 2007; Palop and Mucke, 2009, 2016; Noebels, 2011; Chin and Scharfman, 2013; Vossel et al., 2013). It has been proposed that both the recurrent seizure activity and compensatory homeostatic responses to this seizure activity may interfere with normal neuronal and synaptic functions essential for learning and memory (Leonard and McNamara, 2007; Palop and Mucke, 2009, 2016; Noebels, 2011; Scharfman, 2012a,b; Chin and Scharfman, 2013). Seizures, epileptiform activity, and hippocampal neuronal network hyperactivity were reported in the mild cognitive impairment (MCI) and early stages of AD in humans (Bakker et al., 2012; Vossel et al., 2013). In fact, in MCI and AD patients, cognitive decline began five to seven years earlier in those with epilepsy than in those without, further strengthening the idea of a possible causal association between network hyperexcitability and memory impairment (Vossel et al., 2013). Also, AD patients with subclinical epileptiform activity were found to have an early onset of cognitive decline (Vossel et al., 2013). Additionally, in Aβ-based AD transgenic mice, epileptiform activity and neuronal network hyperexcitability have been reported much before the development of Aβ plaques and overt cognitive impairment (Del Vecchio et al., 2004; Westmark et al., 2010; Bezzina et al., 2015; Kazim et al., 2017; Zott et al., 2019). Also, neural hyperactivity was reported to increase tau release and spread, critical processes in the progression of AD pathology (Pooler et al., 2013; Wu et al., 2016). Neuronal activity was also found to regulate the brain regional vulnerability to Aβ deposition (Bero et al., 2011). These data provide strong evidence of a potential role of neuronal network hyperexcitability in cognitive deficit and disease progression in AD, and further studies in this field may yield potential therapeutic strategies for AD.

### Beneficial effect of levetiracetam, an antiepileptic drug, on cognition in AD patients and mouse models: evidence for the role of neuronal network hyperexcitability in cognitive impairment?

Remarkably, reduction of hippocampal hyperactivity by treatment with an antiepileptic drug, levetiracetam (in low dose), was found to improve memory task performance in MCI patients (Bakker et al., 2012, 2015). Another recent study reported a beneficial effect of low dose levetiracetam in early AD patients by inducing a pattern in brain oscillations of decreased coherence in the lower frequency bands and increased coherence in the higher frequency bands (Musaeus et al., 2017). Additionally, several studies on rodent models of AD and aging have demonstrated beneficial effects of levetiracetam treatment not only on learning and memory impairments but also on disease pathology and disease-associated neurogenic and synaptic failure (Koh et al., 2010; Sanchez et al., 2012; Devi and Ohno, 2013; Shi et al., 2013; Das et al., 2018; Fu et al., 2019).

Targeting hippocampal hyperactivity, peripheral administration of low-dose levetiracetam in aged impaired rats improved cognitive function in two separate hippocampus-dependent spatial reference memory tasks (Koh et al., 2010). Similarly, another study reported that pretraining administration of levetiracetam reduced memory dysfunction in aged C57BL/6 mice in the contextual fear conditioning task (Devi and Ohno, 2013). Acute levetiracetam immediately following training also rescued contextual memory decline in aged mice, however, administration 3 h after training interval had no effect (Devi and Ohno, 2013). These data showed that suppressing hyperexcitability with acute levetiracetam around the time of acquisition or during early consolidation may be sufficient to reverse memory decline associated with aging (Devi and Ohno, 2013).

Levetiracetam was found not only to reduce abnormal spike activity (on subdural EEG recordings) but chronic treatment with levetiracetam also reversed hippocampal remodeling, behavioral abnormalities, synaptic dysfunction, and learning and memory impairments in hAPP-J20 mice (Sanchez et al., 2012). Nonetheless, levetiracetam did not affect Aβ deposition in hAPP-J20 mice, and the behavioral and molecular abnormalities reversed within 35 d after the end of levetiracetam treatment. Contrarily, another study showed that chronic levetiracetam treatment not only alleviated behavioral deficits but also reduced amyloid plaques in APPswe/PS1dE9 transgenic mice (overexpressing the Swedish mutation of APP together with PS1 deleted in exon 9; Shi et al., 2013). Levetiracetam increased Aβ clearance, upregulated Aβ transport and autophagic degradation, and inhibited Aβ generation and suppressed γ-secretase activity (Shi et al., 2013). Another study reported that levetiracetam treatment not only reduced network hypersynchrony in human tau transgenic mice (htau-A152T) but also rapidly and persistently reversed brain dysrhythmia, thus ameliorating network dysfunction (Das et al., 2018). A recent study reported that early seizure activity accelerated depletion of hippocampal neural stem cells and impaired spatial discrimination in hAPP-J20 mice, and treatment with levetiracetam restored neurogenesis and improved performance in a neurogenesis-associated spatial discrimination task in this AD mouse model (Fu et al., 2019).

Overall, the rescue of cognitive dysfunction by antiepileptic drug, levetiracetam, in human AD patients and in aging and AD rodent models, provides an indirect evidence for the role of neuronal network hyperexcitability in memory impairment associated with the disease. Neuronal network hyperexcitability may interfere with encoding or consolidation of memory, and an antiepileptic drug treatment could ameliorate this memory dysfunction by suppressing hyperexcitability.

### Neuronal network hyperexcitability in AD mouse models: roles of Aβ and tau

The role of Aβ in enhancing neuronal network excitability in AD mouse models is well characterized (Palop et al., 2007; Palop and Mucke, 2010a,b, 2016; Noebels, 2011; Scharfman, 2012a,b; Chin and Scharfman, 2013; Born et al., 2014; Bezzina et al., 2015; Born, 2015; Kazim et al., 2017; Zott et al., 2019). However, the role of tau, the other major neuropathological hallmark of AD besides Aβ and a better correlate of cognitive impairment in AD (Nelson et al., 2012), in neuronal network excitability remains unclear with different studies reporting conflicting roles, i.e., enhancement versus suppression (Roberson et al., 2007; García-Cabrero et al., 2013; Holth et al., 2013; Angulo et al., 2017; Hatch et al., 2017; Mondragón-Rodríguez et al., 2018a; Busche et al., 2019). Furthermore, recent studies indicate that experimental models that use expression of both Aβ and tau are more physiologically relevant. Indeed, current understanding of AD pathophysiology is that Aβ initiates a cascade of pathologic events that lead to tau misfolding and aggregation. Ultimately, tau spreads throughout the cortex, resulting in neurodegeneration and cognitive deficits (Busche and Hyman, 2020).

In this paper, we review the relevant literature to date and offer perspectives on the similar versus divergent roles of Aβ and tau in neuronal network excitability in AD.

## Aβ Induces Neuronal Network Hyperexcitability in AD, Even before the Development of Plaques

### Seizures and epilepsy as a co-morbidity in familial AD patients harboring mutations which lead to increased Aβ production

Seizures and epilepsy are a frequent co-morbidity in individuals with EOFAD which is caused by autosomal dominant mutations in APP, PSEN1, or PSEN2 genes, resulting in increased Aβ production and altered Aβ_42_/Aβ_40_ ratio (Noebels, 2011; Guerreiro and Hardy, 2014; Born, 2015). PSEN1 mutations are the most common cause of EOFAD (∼185 mutations PSEN1 mutations identified; Campion et al., 1999; Janssen et al., 2003). Only 33 APP mutations and 13 PSEN2 mutations have been identified as yet (Campion et al., 1999; Janssen et al., 2003; Bekris et al., 2010; Guerreiro and Hardy, 2014). In EOFAD, disease onset is typically at a younger age (<65 years of age) and disease progression is more aggressive as compared with sporadic, late-onset AD.

Seizures have been reported in many PSEN1 mutations carriers. For example, convulsive seizures were described in 37–58% patients with PSEN1 E280A mutation (Larner and Doran, 2006). The S107F mutation, one of the most aggressive PSEN1 mutation pedigrees, was reported to lead to cognitive dysfunction by 26–27 years of age and tonic–clonic seizures in two of the three affected family members (Snider et al., 2005). Patients with other PSEN1 mutations, M146L (Morelli et al., 1998), M223V (Houlden et al., 2001), or L235P (Campion et al., 1996) mutations, developed memory impairment in their 30 s and exhibited seizures and myoclonus.

Mutations in APP and PSEN2, the other two genes that cause familial AD, were also associated with seizures. Seizures were described in 31% (20 out of 64) patients in a case series with N141I PSEN2 mutation (Jayadev et al., 2010). Epileptic seizures were also reported in families with other PSEN2 mutations, for instance, M239V (Marcon et al., 2004) and T430M (Ezquerra et al., 2003) mutations. APP mutations have also been linked with epileptic seizures, for example, T714I (Edwards-Lee et al., 2005), T714A (Lindquist et al., 2008), and V7171G (Kennedy et al., 1993) mutations.

Seizures are also common in patients who carry extra copies of APP. In one study, seizures were reported in 57% of affected individuals with dementia carrying APP duplication (Cabrejo et al., 2006). Seizures and epilepsy are also more frequently observed in DS individuals (who carry APP overexpression by virtue of trisomy 21 and who universally develop AD neuropathological hallmarks and dementia by age 40–55). In one study of 96 down syndrome (DS) cases, 84% were found to develop seizures (Lai and Williams, 1989). In another study of 191 DS adults aged 19–69, 9.4% had epilepsy and the prevalence increased with age; 46% of patients older than 50 had epilepsy (McVicker et al., 1994).

Taken together, these studies suggest that familial forms of AD characterized by abnormal Aβ processing and deposition, a final common pathway in all of these genetic causes of AD, are specifically associated with high occurrence of seizures and epilepsy (Friedman et al., 2012; Born, 2015).

### Neuronal network hyperexcitability in hAPP/Aβ mouse models of AD

Many Aβ-based transgenic mouse models exist that exhibit AD-like behavioral phenotype (Elder et al., 2010; Hall and Roberson, 2012; Webster et al., 2014). Most of the Aβ-based AD transgenic mouse models carry one or more APP mutations found in EOFAD (Campion et al., 1999). Several transgenic APP-overexpression AD mouse models have been found to exhibit neuronal network hyperexcitability (epileptiform activity, behavioral seizure, or increased seizure susceptibility) including Tg2576 (hAPP Swedish mutation, Prp promoter; Hsiao et al., 1996; Westmark et al., 2008, 2010; Corbett et al., 2013; Bezzina et al., 2015; Chan et al., 2015; Duffy et al., 2015; Kam et al., 2016; Ciccone et al., 2019), hAPP-J20 (hAPP Swedish and Indiana mutations, PDGF-β promoter; Palop et al., 2007; Sanchez et al., 2012; Verret et al., 2012; Martinez-Losa et al., 2018), APP23 (hAPP Swedish mutation, Thy-1 promoter; Lalonde et al., 2005), APP23xPS45 (hAPP Swedish and PSEN1 mutations, Thy-1 promoter; Busche et al., 2008, 2012), APdE9 (hAPP Swedish and PSEN1:deltaE9 mutations, Prp promoter; Minkeviciene et al., 2009; Ziyatdinova et al., 2011, 2015; Gurevicius et al., 2013; Nygaard et al., 2015; Reyes-Marin and Nuñez, 2017), APP/TTA (hAPP Swedish and Indiana mutations, CamKIIα promoter; Born et al., 2014), TgCRND8 (hAPP Swedish and Indiana mutations, Prp promoter; Jolas et al., 2002; Del Vecchio et al., 2004), 3xTg-AD (hAPP Swedish, htau P301L, and PSEN1:M146V mutations, Thy-1 promoter; Davis et al., 2014; Nygaard et al., 2015; Frazzini et al., 2016; Kazim et al., 2017), and 5XFAD (hAPP Swedish, Florida, and London mutations and two PSEN1 (M146L, L286V) mutations, Thy-1 promoter (Siwek et al., 2015). Recent studies suggest the existence of a feed-forward induction loop between Aβ and neuronal network hyperexcitability as it was shown that neural activity modulates Aβ production (Cirrito et al., 2008; Bero et al., 2011). Table 1 summarizes main findings of studies evaluating neuronal network hyperexcitability in Aβ mouse models of AD.

**Table 1:** Studies evaluating neuronal network excitability in hAPP/Aβ mouse models of AD

Author(s) and publication year	Mouse model/transgene(s)/promoter	Age/stage of pathology	Neuronal network excitability status	Experimental paradigm/neuronal network excitability observation(s)
Studies assessing neuronal network hyperexcitability in hAPP/Aβ mice at advanced stages of Aβ plaque pathology and cognitive impairment				
Palop et al. (2007)	hAPP-J20 (hAPP Swedish and Indiana) PDGF-β promoter	4–7 months, Aβ plaques deposition, cognitive impairment, synaptic deficit	Increased	Experimental paradigm: *in vivo* chronic video EEG recordings; PTZ-induced seizure susceptibility; *in vitro* mIPSCs and fEPSPs recordings. Findings: frequent epileptiform activity including spikes and SWDs and increased PTZ-induced seizure susceptibility in hAPP-J20 mice. Reduced LTP and PPF in hippocampal perforant pathway in hAPP-J20 mice slices. Increased dentate granule cells mIPSCs frequency in hAPP-J20 mice. Remodeling of inhibitory circuits and altered NPY expression in dentate gyrus of hAPP-J20 mice.
Verret et al. (2012)	hAPP-J20 (hAPP Swedish and Indiana) PDGF-β promoter	4–7 months, Aβ plaques deposition, cognitive impairment, synaptic deficit	Increased	Experimental paradigm: *in vivo* chronic video EEG recordings. Findings: spontaneous epileptiform discharges observed during reduced γ oscillatory activity (generated by inhibitory PV cells) in hAPP-J20 mice. Decreased levels of the interneuron-specific and PV cell-predominant voltage-gated sodium channel subunit Na_v_1.1. Restoring Na_v_1.1 level in hAPP-J20 mice increased inhibitory synaptic activity and γ oscillations and reduced hyperexcitability and cognitive deficits.
Sanchez et al. (2012)	hAPP-J20 (hAPP Swedish and Indiana) PDGF-β promoter	4–6 months, Aβ plaques deposition, cognitive impairment, synaptic deficit	Increased	Experimental paradigm: *in vivo* chronic video EEG recordings; fEPSPs in acute hippocampal slices. Findings: spontaneous epileptiform activity in hAPP-J20 mice. Chronic treatment with levetiracetam reversed abnormal spiking activity, hippocampal remodeling, behavioral abnormalities, synaptic dysfunction, and deficits in learning and memory in hAPP-J20 mice.
Martinez-Losa et al. (2018)	hAPP-J20 (hAPP Swedish and Indiana) PDGF-β promoter	7–8 months, Aβ plaques deposition, cognitive impairment, synaptic deficit	Increased	Experimental paradigm: *in vivo* EEG recordings in freely moving mice. Findings: epileptiform spikes on cortical EEG in hAPP-J20 mice. Na_v_1.1-overexpressing, interneuron transplants enhanced reduced network hypersynchrony and improved cognitive functions in hAPP-J20 mice.
Minkeviciene et al. (2009)	APdE9 (hAPP Swedish and PSEN1: deltaE9) Prp promoter	3 and 4.5 months, substantial number Aβ plaques observed in cortex, hippocampus, and amygdala	Increased	Experimental paradigm: *in vivo* video EEG recordings; patch clamp electrophysiology; extracellular field recordings in brain slices. Findings: unprovoked seizures in APdE9 mice. Hyperexcitability in neocortical layer 2/3 pyramidal cells in APdE9 mice on patch clamp recordings. Aβ protofibrils induced neuronal network hyperexcitability in acute brain slices.
Ziyatdinova et al. (2011)	APdE9 (hAPP Swedish and PSEN1: deltaE9) Prp promoter	4–5 months, Aβ plaques in the neocortex and hippocampus	Increased	Experimental paradigm: *in vivo* video EEG recordings. Findings: spontaneous electrographic epileptiform discharges. Antiepileptic drugs that block sodium chan-nels, including carbamazepine, phenytoin, and valproic acid suppressed epileptiform activity in APdE9 mice with increased amyloid pathology.
Gurevicius et al. (2013)	APdE9 (hAPP Swedish and PSEN1: deltaE9) Prp promoter	4 months; Aβ plaques in the neocortex and hippocampus	Increased	Experimental paradigm: *in vivo* EEG recordings from the hippocampus, cerebral cortex, and thalamus during movement, quiet waking, non-rapid eye movement sleep, and REM sleep. Findings: cortical EEG power was higher in APdE9 mice than in WT mice over a broad frequency range (5–100 Hz) and during all 4 behavioral states. Thalamic EEG power was also increased but in a narrower range (10–80 Hz). While power and θ–γ modulation were preserved in the APdE9 hippocampus, REM sleep-related phase shift of θ–γ modulation was altered.
Ziyatdinova et al. (2015)	APdE9 (hAPP Swedish and PSEN1: deltaE9) Prp promoter	4–5 months, Aβ plaques in the neocortex and hippocampus	Increased	Experimental paradigm: *in vivo* video EEG recordings. Findings: spontaneous epileptiform discharges. Antiepileptic drug valproic acid reduced the amount of epileptiform activity, but the effect disappeared after treatment discontinuation.
Nygaard et al. (2015)	APdE9 (hAPP Swedish and PSEN1: deltaE9) Prp promoter	10 months, Aβ plaques in the cortex and hippocampus	Increased	Experimental paradigm: *in vivo* video EEG recordings. Findings: epileptiform activity in the form of SWDs in APdE9 mice. SWDs correlated with spatial memory impairment in these mice. Brivaracetam (a chemical analog of levetiracetam) reduced SWDs and reversed memory impairments in in APdE9 mice.
Reyes-Marin and Nuñez (2017)	APdE9 (hAPP Swedish and PSEN1: deltaE9) Prp promoter	4–9 months, Aβ plaques in the cortex and hippocampus	Increased	Experimental paradigm: *in vivo* video EEG recordings; PTZ-induced seizure susceptibility. Findings: higher incidence of epileptiform-like discharges, i.e., seizure events (interictal spikes, sharp waves, or polyspikes) in APdE9 than in the controls. Also, APdE9 mice showed a lower latency to PTZ-evoked seizure events than in the control animals. A correlation was also found between the frequency of epileptiform-like discharges and the number of Aβ plaques.
Busche et al. (2008)	APP23xPS45 (hAPP_751_ Swedish and PSEN1-Gly384→Ala384, G384A) Thy-1 promoter	8–10 months, Aβ plaques	Increased	Experimental paradigm: *in vivo* two-photon Ca^2+^ imaging of neurons in layer 2/3 of the cortex. Findings: clusters of hyperactive neurons were found in the vicinity of Aβ plaques.
Busche et al. (2012)	APP23xPS45 (hAPP_751_ Swedish and PSEN1-Gly384→Ala384, G384A) Thy-1 promoter	6–7 months, Aβ plaques	Increased	Experimental paradigm: *in vivo* two-photon Ca^2+^ imaging of CA1 pyramidal neurons in the hippocampus. Findings: hyperactive neurons were found to be located exclusively in the vicinity of Aβ plaques in the hippocampus of transgenic mice.
Lalonde et al. (2005)	APP23 (hAPP_751_ Swedish) Thy-1 promoter	24 months, Aβ plaques	Increased	Experimental paradigm: behavioral seizures evaluation. Findings: 41% of APP23 mice exhibited tonic-clonic seizures; 24% displayed myoclonic jumping.
Jolas et al. (2002)	TgCRND8 (hAPP_695_ Swedish and Indiana) Prp promoter	5 months, Aβ plaques	Increased	Experimental paradigm: *in vitro* hippocampal electrophysiology recordings; evoked EPSCs and IPSCs; PTZ-induced seizure threshold. Findings: increased synaptic excitability and increased maximum amplitude of evoked mEPSCs; consistently lower dose of PTZ was required to elicit myoclonic activity (preseizure signs) in TgCRND8 mice compared with controls.
Siwek et al. (2015)	5XFAD (hAPP Swedish, Florida, and London plus PSEN1: M146L and L286V) Thy-1 promoter	16.5 months, Aβ plaques throughout hippocampus and cortex	Increased	Experimental paradigm: *in vivo* video EEG recordings from the cortex and the hippocampus. Findings: aberrant hyperexcitability in 5×FAD mice evidenced as ictal-like discharges, such as spikes, polyspikes, and spike-waves.
Davis et al. (2014)	3xTg-AD (hAPP Swedish. htau P301L, and hPSEN1: M146V) Thy-1.2 promoter	17–18 months, Aβ plaques	Increased	Experimental paradigm: *in vivo* hippocampal electrophysiology recordings. Findings: increased synaptic excitability in DG and CA1.
Nygaard et al. (2015)	3xTg-AD (hAPP Swedish. htau P301L, and hPSEN1: M146V) Thy-1.2 promoter	8–10 months, Aβ plaques, cognitive impairment	Increased	Experimental paradigm: *in vivo* video EEG recordings from the cortex. Findings: SWDs in 3×Tg-AD mice which correlated with spatial memory impairments.
Chan et al. (2015)	Tg2576 (hAPP Swedish), Prp promoter	12–14 months, Aβ plaques, cognitive impairment	Increased	Experimental paradigm: electrical amygdala kindling with implanted electrodes and behavioral seizures evaluation. Findings: Tg2576 mice exhibited increased susceptibility to kindling and seizure-associated death.
Studies evaluating neuronal network hyperexcitability in hAPP/Aβ mice at early stages of Aβ pathology before plaque deposition and/or cognitive impairment				
Westmark et al. (2008)	Tg2576 (hAPP Swedish), Prp promoter	2 months, before Aβ plaques deposition and cognitive impairment	Increased	Experimental paradigm: PTZ-induced seizure susceptibility assessment. Findings: increased susceptibility to PTZ-induced seizures in Tg2576 mice.
Westmark et al. (2010)	Tg2576 (hAPP Swedish), Prp promoter	3 weeks, before Aβ plaques deposition and cognitive impairment	Increased	Experimental paradigm: audiogenic seizure susceptibility evaluation. Findings: increased susceptibility to audiogenic seizures in Tg2576 mice as compared with WT controls. The audiogenic seizure susceptibility in Tg2576 mice could be suppressed by passive immunization with an anti-APP/Aβ antibody or by blockade of mGluR5 with the selective antagonist, MPEP.
Corbett et al. (2013)	Tg2576 (hAPP Swedish), Prp promoter	5–7 months, before Aβ plaques deposition	Increased	Experimental paradigm: *in vivo* EEG recordings. Findings: presence of SWDs and abnormal EEG patterns in Tg2576 mice; these mice also exhibited longer durations of higher frequency brain activity, suggesting increased synchrony.
Bezzina et al. (2015)	Tg2576 (hAPP Swedish), Prp promoter	1.5–2 months, before Aβ plaques deposition and cognitive impairment	Increased	Experimental paradigm: electrical amygdala kindling with implanted electrodes and behavioral seizures evaluation. Findings: Tg2576 mice exhibited increased susceptibility to kindling and seizure-associated death.
Duffy et al. (2015)	Tg2576 (hAPP Swedish), Prp promoter	2–4 months, prior to Aβ plaques deposition; soluble Aβ_40_ and Aβ_42_ detectable; impairment in object location, an EC-dependent cognitive task.	Increased	Experimental paradigm: *ex vivo* EC recordings. Findings: increased excitability in EC recordings in slices from Tg2576 mice.
Kam et al. (2016)	Tg2576 (hAPP Swedish), Prp promoter	5 weeks, prior to Aβ plaques deposition and cognitive impairment	Increased	Experimental paradigm: *in vivo* video EEG recordings. Findings: synchronized large amplitude potentials resembling interictal spikes in epilepsy were observed in Tg2576 mice.
Ciccone et al. (2019)	Tg2576 (hAPP Swedish), Prp promoter	3 months, before Aβ plaques deposition	Increased	Experimental paradigm: extracellular fEPSP activity elicited by the proconvulsant drug 4-aminopyridine (4-AP) in acute hippocampal slices from 3-month-old WT and Tg2576 slices. Findings: significantly higher number of electrical discharges, occurring with similar amplitude but shorter intervals, was observed in Tg2576 in comparison to WT hippocampal slices after 4-AP application.
Del Vecchio et al. (2004)	TgCRND8 (hAPP_695_ Swedish and Indiana) Prp promoter	6–8 weeks, before Aβ plaques deposition	Increased	Experimental paradigm: PTZ-induced seizure susceptibility evaluation. Findings: increased susceptibility to PTZ-induced seizures in TgCRND8 mice.
Fontana et al. (2017)	PS2APP (hAPP Swedish and hPSEN2: N141I) Thy1 (hAPP) and Prp(hPSEN2) promoters	3 months, before Aβ plaques deposition and cognitive impairment	Increased	Experimental paradigm: *in vivo* spontaneous LFPs in DG. Findings: network hypersynchronicity was observed in the DG of PS2APP mice.
Busche et al. (2012)	APP23xPS45 (hAPP_751_ Swedish and PSEN1-Gly384→Ala384, G384A) Thy-1 promoter	1.5 months, before Aβ plaques deposition and cognitive impairment	Increased	Experimental paradigm: *in vivo* two-photon calcium imaging of the hippocampal CA1 neurons. Findings: selective increase in hyperactive neurons in hippocampus of APP23xPS45 mice before Aβ plaques deposition suggesting that soluble species of Aβ may underlie this impairment. Acute treatment with the γ-secretase inhibitor LY-411575 reduced soluble Aβ levels and rescued the neuronal dysfunction.
Davis et al. (2014)	3xTg-AD (hAPP Swedish. htau P301L, and hPSEN1: M146V) Thy-1.2 promoter	4–6 months, before Aβ plaques deposition	Increased	Experimental paradigm: *in vivo* hippocampal electrophysiology recordings. Findings: synaptic hyperexcitability in DG and CA1.
Kazim et al. (2017)	3xTg-AD (hAPP Swedish. htau P301L, and hPSEN1: M146V) Thy-1.2 promoter	3 weeks, before Aβ plaques deposition and cognitive impairment	Increased	Experimental paradigm: audiogenic seizure susceptibility; *ex vivo* hippocampal CA3 intracellular recordings after GABA_A_ blockade with bicuculline. Findings: increased audiogenic seizure susceptibility and prolonged epileptiform discharges after bicuculline application in hippocampal CA3 intracellular recordings in 3×Tg-AD mice.
Fu et al. (2019)	hAPP-J20 (APP Swedish and Indiana) PDGF-β promoter	1 and 2 months, before Aβ plaques deposition and cognitive impairment	Increased	Experimental paradigm: *in vivo* EEG recordings. Findings: epileptic spikes at 1 month of age with robust seizure activity at 2 months of age.

4-AP, 4-aminopyridine; Aβ, amyloid β; DG, dentate gyrus; EC, entorhinal cortex; EEG, electroencephalogram; fEPSPs, field EPSPs; hAPP, human amyloid β precursor protein; hPSEN, human presenilin; LFPs, local field potentials; LTP. Long-term potentiation; mEPSCs, miniature EPSCs; mGluR5, metabotropic glutamate receptor 5; mIPSCs, miniature IPSCs; MPEP, 2-methyl-6-(phenylethynyl)pyridine hydrochloride; PDGF, platelet-derived growth factor; Prp, prion protein; PTZ, phenylenetetrazole; PV, parvalbumin; REM, rapid eye movement; SWDs, spike-wave discharges; WT, wild type.

### Neuronal network hyperexcitability in hAPP/Aβ mice at advanced stages of disease pathology and cognitive impairment

**Figure 1.**
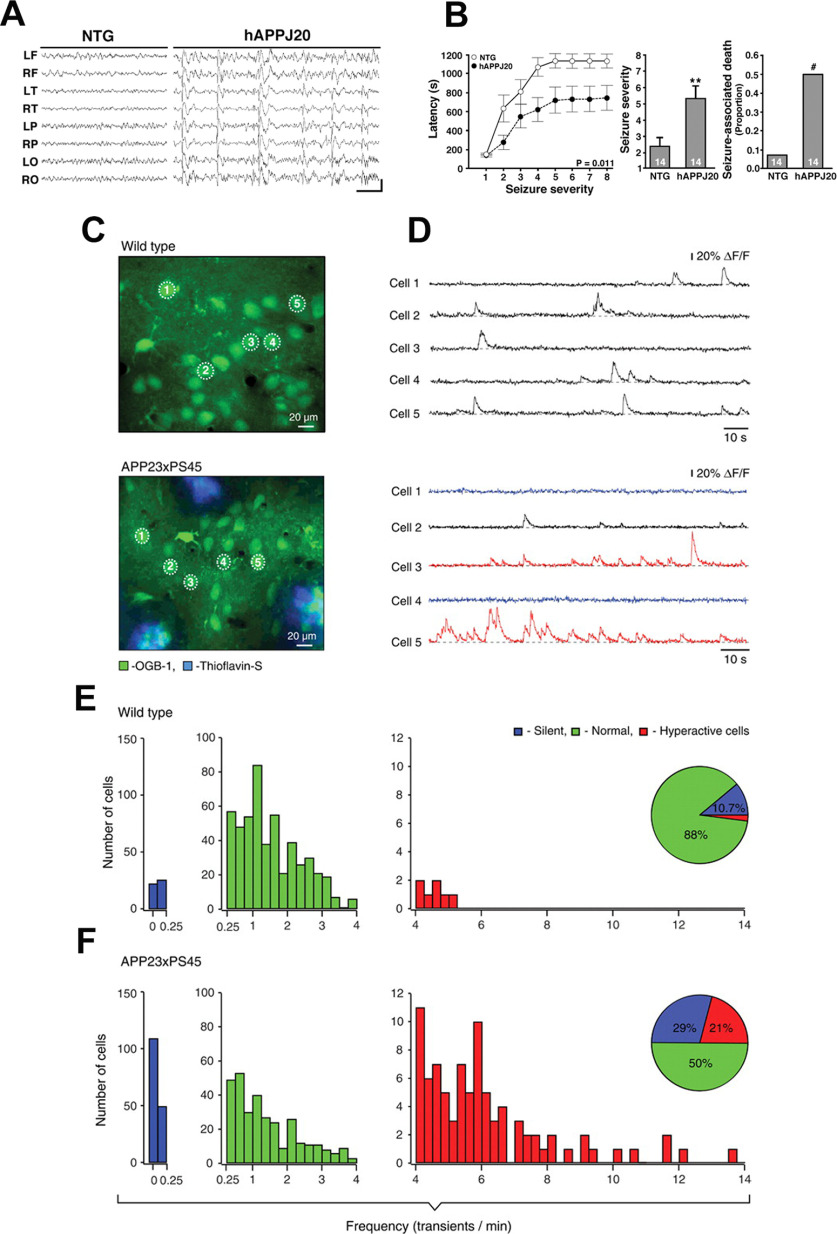
Neuronal network hyperexcitability at advanced stages of pathology in hAPP/Aβ mouse models of AD. ***A***, ***B***, Aberrant synchronous neuronal network activity, spontaneous nonconvulsive seizures, and increased susceptibility to PTZ-induced seizures in four- to seven-month-old hAPP-J20 mice. Reproduced from Palop et al. (2007) with permission from Elsevier. ***A***, Chronic cortical EEG recordings performed in freely moving, untreated hAPP-J20 mice, and non-transgenic (NTG) controls. L, left; R, right; F, frontal; T, temporal; P, parietal; O, posterior-parietal, indicate the position of recording electrodes. In contrast to NTG mice, which showed normal EEG activity (left), hAPP-J20 mice exhibited frequent (5–50/min) generalized cortical epileptiform (interictal) spike discharges (right). Calibration: 1 s and 400 mV. ***B***, Mice were injected intraperitoneally with PTZ (GABA_A_ antagonist), behavior was videorecorded, and seizure severity was scored off-line. Compared with NTG controls, hAPP-J20 mice had shorter latencies to reach a given seizure severity (left), greater overall seizure severity (center), and more seizure-associated deaths (right); ∗∗*p* < 0.01 versus NTG by Student’s *t* test; #*p* < 0.05 by Fisher’s exact test. Quantitative data represent mean ± SEM. ***C–F***, Clusters of hyperactive neurons near amyloid plaques in APP23xPS45 mice. *In vivo* two-photon calcium imaging from layer 2/3 cortical neurons. Reproduced with permission from Busche et al. (2008). ***C***, ***D***, Spontaneous Ca^2+^ transients (***D***) recorded *in vivo* in the corresponding neurons of the frontal cortex shown in ***C*** in a WT (top) and a APP23xPS45 (bottom) mouse. Traces in ***D***, bottom, are color-coded to mark neurons that were either inactive during the recording period (blue) or showed an increased frequency of Ca^2+^ transients (red). ***E***, ***F***, Histograms showing the frequency distribution of Ca^2+^ transients in WT and APP23xPS45 mice (in both cases *n* = 564 cells). There is a substantial increase in the amount of silent and hyperactive neurons in APP23xPS45 mice. (Insets) Pie charts showing the relative proportion of silent, normal, and hyperactive neurons in WT (*n* = 10) and APP23xPS45 (*n* = 20) mice.

In 2007, a landmark study was published which showed the presence of spontaneous epileptiform discharges in hAPP-J20 mice [harboring hAPP Swedish (KM670/671NL) and hAPP Indiana (V717F) mutations; transgene expression being driven by the PDGF-β promoter; Fig. 1*A*,*B*; Palop et al., 2007]. The study brought into focus the question that AD transgenic mice may be undergoing spontaneous intermittent episodes of generalized non-convulsive seizures without the investigators being aware of the phenomenon. Palop et al. (2007) continually monitored neuronal activity in cortical and hippocampal networks by video EEG recordings in four- to seven-month-old hAPP-J20 mice, which have Aβ plaques in the hippocampus and neocortex and demonstrate behavioral and synaptic deficits. They reported the presence of frequent epileptiform activity including spikes and sharp waves, and intermittent unprovoked seizures involving neocortex and hippocampus that were not accompanied by tonic or clonic motor activity (Fig. 1*A*; Palop et al., 2007). Additionally, increased susceptibility to phenylenetetrazole (PTZ)-induced seizures was observed in hAPP-J20 mice as compared with WT controls (Fig. 1*B*; Palop et al., 2007). Also, epileptic activity led to compensatory inhibitory remodeling of the hippocampal circuitry to counteract network activity imbalances (Palop et al., 2007). GABAergic sprouting, enhanced synaptic inhibition, and synaptic plasticity deficits in the dentate gyrus were also observed in hAPP-J20 mice (Palop et al., 2007). It was proposed that both the recurrent seizure activity and compensatory homeostatic responses to this seizure activity may interfere with normal neuronal and synaptic functions essential for learning and memory (Leonard and McNamara, 2007; Palop et al., 2007; Palop and Mucke, 2009, 2010a,b, 2016; Noebels, 2011; Scharfman, 2012a,b; Chin and Scharfman, 2013).

A subsequent study from the same group further confirmed the presence of spontaneous epileptiform activity and network hypersynchrony on cortical EEG recordings in four- to seven-month-old hAPP-J20 mice (Verret et al., 2012). Primarily, the spontaneous epileptiform discharges were observed during reduced γ oscillatory activity (important for learning and memory). As this oscillatory rhythm is generated by inhibitory PV cells, it was hypothesized that network dysfunction in hAPP-J20 mice might arise from impaired PV cells (Verret et al., 2012). In fact the study found that hAPP-J20 mice and AD patients had decreased levels of the interneuron-specific and PV cell-predominant voltage-gated sodium channel subunit Na_v_1.1 (Verret et al., 2012). Restoring Na_v_1.1 level in hAPP-J20 mice by Na_v_1.1-BAC expression increased inhibitory synaptic activity and γ oscillations and reduced hyperexcitability, cognitive deficits, and premature mortality. Thus, it was concluded that reduced Na_v_1.1 levels and PV cell dysfunction critically mediate abnormalities in oscillatory brain rhythms, network synchrony, and memory in hAPP-J20 mice, and possibly in AD (Verret et al., 2012). A recent study further corroborated this as Na_v_1.1-overexpressing, interneuron transplants (derived from the embryonic medial ganglionic eminence) were found to enhance behavior-dependent γ oscillatory activity, reduce network hypersynchrony, and improve cognitive functions in hAPP-J20 mice (Martinez-Losa et al., 2018).

Another study in four- to six-month-old hAPP-J20 mice provided evidence for the causal relationship between neuronal network hyperexcitability and cognitive dysfunction (Sanchez et al., 2012). The antiepileptic drug levetiracetam was found to effectively reduce abnormal spike activity detected by EEG (Sanchez et al., 2012). Chronic treatment with levetiracetam also reversed hippocampal remodeling, behavioral abnormalities, synaptic dysfunction, and deficits in learning and memory in hAPP-J20 mice (Sanchez et al., 2012). These data supported the hypothesis that aberrant network activity contributes causally to synaptic and cognitive deficits in Aβ mice. Nonetheless, it is imperative to note here that behavioral and molecular abnormalities recurred within 35 d after end of levetiracetam treatment in hAPP-J20 mice (Sanchez et al., 2012), suggesting that a chronic persistent treatment of network hyperexcitability may be required to ameliorate AD-associated cognitive dysfunction.

Previously, in six- to eight-month-old double transgenic APP23xPS45 mice [harboring the 751 isoform of hAPP Swedish (KM670/671NL) and PSEN1 (Gly384→Ala384, G384A) mutations under the control of Thy-1 promoter; cognitively impaired at this age], *in vivo* two-photon Ca^2+^ imaging in layer 2/3 cortical neurons revealed clusters of hyperactive neurons near Aβ plaques (Fig. 1*C–F*; Busche et al., 2008). While the study found a decrease in neuronal activity in 29% of layer 2/3 cortical neurons, remarkably 21% of neurons displayed an unexpected increase in the frequency of spontaneous Ca^2+^ transients (Busche et al., 2008). These hyperactive neurons were found exclusively in the vicinity of the plaques of Aβ-depositing APP23xPS45 mice (Busche et al., 2008). It was reported that not only did hyperactive neurons fire more frequently, they also did this in a correlated manner, thus increasing the risk for seizure-like activity (Busche et al., 2008). The hyperactivity appeared to be because of a relative decrease in synaptic inhibition (Busche et al., 2008). The study suggested that an anatomic remodeling of both excitatory and inhibitory synaptic inputs gave rise to the observed changes in neuronal function (Busche et al., 2008), this was in congruence with the finding of inhibitory interneuron remodeling reported in the hippocampus of amyloid plaques bearing hAPP-J20 mice demonstrating spontaneous epileptiform activity (Palop et al., 2007). Another study from the same group (Busche et al., 2012) reported the presence of hyperactive neurons near Aβ plaques in the hippocampus in six- to seven-month-old APP23xPS45 mice. A marked increase in the fractions of both silent and hyperactive neurons was observed in the hippocampus of plaque depositing APP23xPS45 mice (Busche et al., 2012), as previously also found in the cortex (Busche et al., 2008). Also, the hyperactive neurons were found to be located exclusively in the vicinity of plaques in transgenic mice, whereas both silent and normal neurons were distributed throughout the hippocampus (Busche et al., 2012). A recent study from the same group employing *in vivo* two-photon Ca^2+^ imaging reported that hyperactivation in Aβ mouse models is initiated by the suppression of glutamate reuptake (Zott et al., 2019). The astroglial excitatory amino-acid transporter 2 (EAAT2; also termed GLT-1 in mice) is the predominant glutamate transporter in mammalian brain, being responsible for over 90% of glutamate uptake (Danbolt et al., 1992; Haugeto et al., 1996). Aβ was found to interfere with EAAT2-mediated glutamate uptake, thus providing a mechanism for Aβ-mediated neuronal network hyperexcitability in AD (Li et al., 2009; Zott et al., 2019).

We have reported that treatment with the glutamate modulator riluzole, which has been shown to increase EAAT2 expression (Banasr et al., 2010; Hunsberger et al., 2015, 2016) besides other mechanisms of actions, can prevent age-related cognitive decline through clustering of dendritic spines (Pereira et al., 2014), strengthening neural communication (Govindarajan et al., 2006; Larkum and Nevian, 2008). Furthermore, we have shown that riluzole rescues age and AD-gene expression profile (Pereira et al., 2017). More recently, we have published that riluzole prevents hippocampal-dependent spatial memory decline in an early-onset and aggressive mouse model of AD (5XFAD) and reversed many of the gene expression changes in immune pathways (Okamoto et al., 2018), and specifically microglia-related genes thought to be critical mediators of AD pathophysiology (Streit, 2004; Butovsky et al., 2014; Colonna and Wang, 2016), including a recently identified unique population of disease-associated microglia (DAM; Keren-Shaul et al., 2017).

In a study of 3- and 4.5-month-old APdE9 mice [harboring hAPP Swedish (KM670/671NL) and PSEN1:deltaE9 mutations; transgene expression being driven by the Prp promoter], neuronal hyperexcitability culminating in epileptiform activity in the presence of Aβ plaques was reported (Minkeviciene et al., 2009). In video EEG recordings, at least one unprovoked seizure was detected in 65% of APdE9 mice, of which 46% had multiple seizures and 38% had a generalized seizure, whereas none of the WT mice had seizures (Minkeviciene et al., 2009). In a subset of APdE9 mice, seizure phenotype was associated with a loss of calbindin-D28k immunoreactivity in dentate granular cells and ectopic expression of neuropeptide Y (NPY) in mossy fibers (Minkeviciene et al., 2009). In APdE9 mice, persistently decreased resting membrane potential in neocortical layer 2/3 pyramidal cells and dentate granule cells was observed which could be responsible for neuronal network hyperexcitability as identified by patch-clamp electrophysiology (Minkeviciene et al., 2009). Bath application of Aβ protofibrils was found to induce significant membrane depolarization of pyramidal cells and increased the activity of excitatory cell populations as measured by extracellular field recordings in the rodent brain slices, confirming the pathogenic significance of Aβ in neuronal network hyperexcitability (Minkeviciene et al., 2009). Another study in four-month-old APdE9 mice further confirmed increased cortical and thalamic excitability (Gurevicius et al., 2013). A subsequent study demonstrated that sodium channel blocking antiepileptic drugs (carbamazepine, valproic acid, or phenytoin) could suppress epileptiform activity in APdE9 mice with increased amyloid pathology (Ziyatdinova et al., 2011). Another study later found that while valproic acid treatment of APdE9 mice, at the stage when amyloid plaques are beginning to develop and epileptiform activity is detected, reduced the amount of epileptiform activity, but the effect disappeared after treatment discontinuation, and no consistent long-term effects were observed (Ziyatdinova et al., 2015). This is in congruence with the data from hAPP-J20 mice where abnormalities returned after discontinuation of levetiracetam treatment, as mentioned earlier (Sanchez et al., 2012). Epileptiform-like discharges, i.e., seizure-related events consisting of interictal spikes, sharp wave discharges or polyspikes were also observed in cortical EEG recordings of four- to nine-month-old APdE9 mice (Reyes-Marin and Nuñez, 2017). Also, a lower latency to PTZ-evoked seizure events was found in APdE9 mice compared with WT controls (Reyes-Marin and Nuñez, 2017). Importantly, a correlation between the frequency of epileptiform-like discharges and the number of Aβ plaques was reported (Reyes-Marin and Nuñez, 2017). Another study also reported the presence of epileptiform activity in the form of spike wave discharges in 8- to 10-month-old APdE9 mice; spike wave discharges correlated with spatial memory impairment in these mice (Nygaard et al., 2015). Interestingly, while antiepileptics ethosuximide and brivaracetam (a chemical analog of levetiracetam) both reduced spike-wave discharges in APdE9 mice, brivaracetam, but not ethosuximide, reversed impairments in spatial memory (Nygaard et al., 2015).

Several other studies in hAPP/Aβ mouse models of AD have identified enhanced seizure susceptibility and/or spontaneous epileptiform activity at advanced stages of the Aβ pathology and cognitive deficit. Increased seizure activity was found in 24-month-old APP23 mice [harboring the 751 isoform of hAPP Swedish (KM670/671NL) mutation under the control of Thy-1 promoter] with extensive Aβ plaque pathology (Lalonde et al., 2005). Increased synaptic excitability and increased maximum amplitude of evoked miniature EPSCs (mEPSCs) was reported in the hippocampus of Aβ plaques bearing 20-week-old TgCRND8 mice [hAPP695 with the Swedish mutation (KM670/671NL) and Indiana mutation (V717F) under the control of the hamster prion (PrP) gene promoter; thioflavin S-positive amyloid deposits at three months; dense cored plaques and neuritic pathology by five months; Jolas et al., 2002]. Cortical hyperexcitability was also reported in ∼72-week (16.5-month)-old 5XFAD mice [harboring three hAPP mutations: Swedish (KM670/671NL), Florida (I716V), and London (V717I) mutations and two PSEN1 (M146L, L286V) mutations under the control of Thy-1 promoter; plaques are found throughout the hippocampus and cortex by six months; Siwek et al., 2015]. Employing *in vivo* electrophysiology, increased hippocampal excitability was reported in 17- to 18-month-old 3xTg-AD mice [harboring hAPP Swedish (KM670/671NL), hPSEN1 M146V, and htau P301L mutations under control of Thy1.2 promoter; these mice develop Aβ plaques and NFTs-like pathologies in a progressive and age-dependent manner, starting at ∼9 and ∼12 months; Davis et al., 2014]. Another study in 8- to 10-month-old 3xTg-AD mice reported the presence of spike wave discharges which correlated with spatial memory impairment in these mice (Nygaard et al., 2015). An increased susceptibility to kindling and seizure-associated death was also reported in aged (12- to 14-month-old) Tg2576 mice [harboring hAPP Swedish mutation (KM670/671NL), under the control of prion protein promoter; numerous parenchymal Aβ plaques are evident by 11–13 months of age; Chan et al., 2015].

### Early-onset neuronal network hyperexcitability in Aβ-based mouse models of AD, much before Aβ plaques and overt cognitive impairment: the role of intraneuronal hAPP/Aβ and soluble Aβ

**Figure 2.**
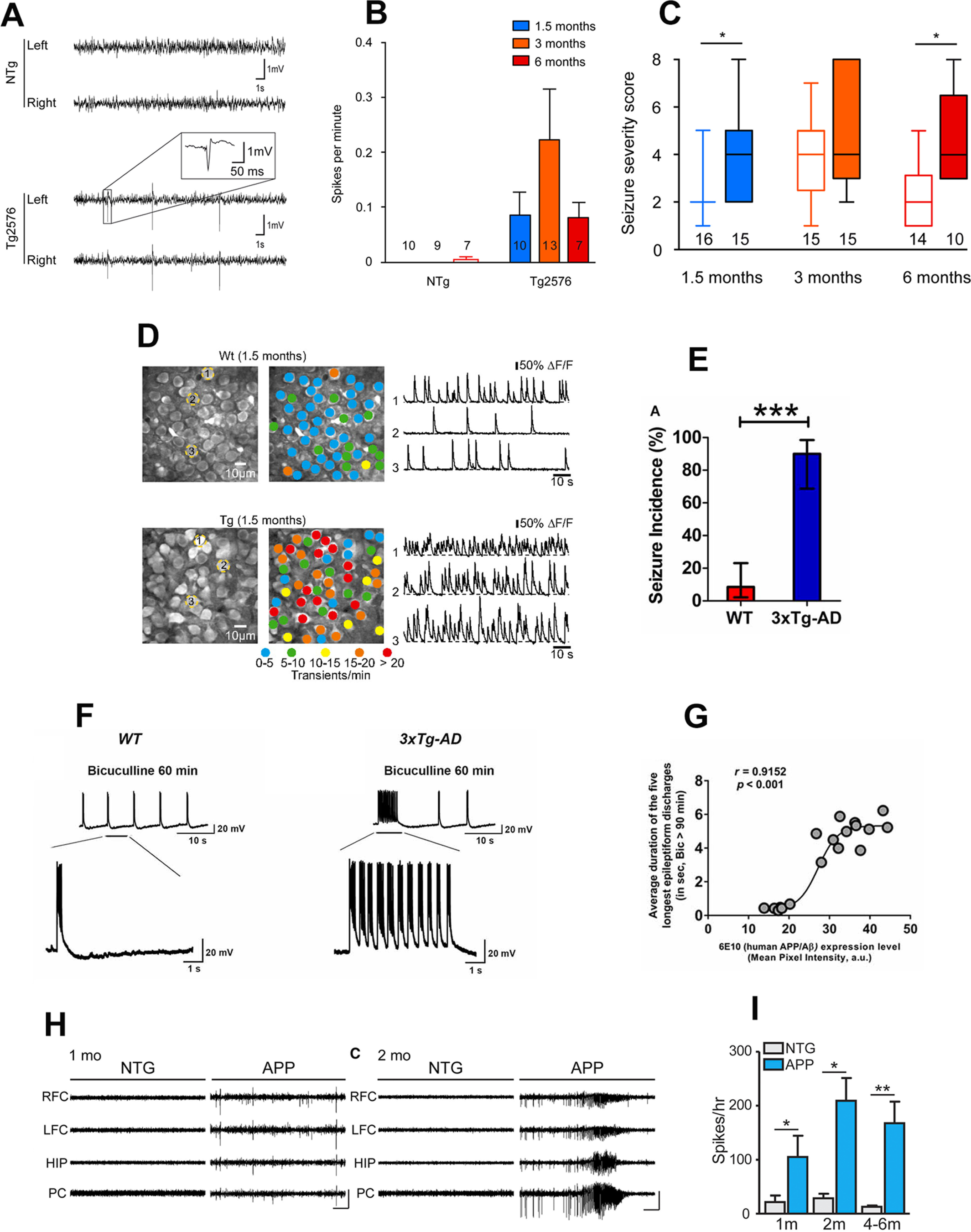
Early-onset neuronal network hyperexcitability in hAPP/Aβ mouse models of AD. ***A–C***, Tg2576 mice exhibit spontaneous epileptiform activity and high susceptibility to pharmacologically induced seizures as young as 1.5 months of age. Reproduced with permission from Bezzina et al. (2015). ***A***, Representative EEG traces from non-transgenic (NTg; top) and Tg2576 (bottom) mice from left and right parietal cortices. Note that only transgenic animals displayed sharp, high-voltage spikes that characterize epileptiform activity (inset). ***B***, Quantitative analysis of the frequency of interictal spikes (mean ± SEM). Two-way ANOVA shows a significant genotype effect (*p* = 0.013) but no age effect (*p* = 0.4091) and no interaction (*p* = 0.3865). Numbers over the horizontal axis indicate the number of mice used in each experimental group. ***C***, Seizure severity score of 1.5-, 3-, and 6-month-old Tg2576 male mice and NTg age-matched littermates. Whiskers boxes represent the interquartile distribution. Number of mice in each group is indicated below the boxes. Tg2576 mice exhibit more severe seizures than NTg at 1.5 and 6 months of age (Dunn’s tests: *p* < 0.05 for Tg2576 vs NTg at 1.5 and 6 months old). Note that only transgenic animals exhibit lethal seizures. Numbers over the horizontal axis indicate the number of mice used in each experimental group. ***D***, Early hyperactivity of hippocampal neurons of 1.5-month-old APP23xPS45 mice (an age when no plaques are detectable). Reproduced with permission from Busche et al. (2012). Left, CA1 neurons imaged *in vivo* in a WT and a transgenic mouse, respectively. Center, Activity maps in which hue is determined by the frequency of spontaneous Ca^2+^ transients, overlaid with the anatomic image (left). Right, Spontaneous Ca^2+^ transients of the corresponding neurons marked (left). ***E–G***, Early-onset seizure susceptibility and epileptiform activities in three-week-old 3xTg-AD mice (much before plaques and overt cognitive impairment). Reproduced with permission from Kazim et al. (2017). ***E***, Incidence of convulsive seizures after audiogenic stimulation was markedly higher in three-week-old 3xTg-AD mice (blue bar) compared with WT mice (red bar). The data are presented as percent incidence with 95% confidence interval and compared using exact logistic regression stratified by litter; ***p < 0.001, compared with WT. WT (*n* = 35) and 3xTg-AD (*n* = 20) mice. ***F***, Ictal-like epileptiform discharges in CA3 pyramidal cells of hippocampal slices from three-week-old 3xTg-AD mice. Left, CA3 intracellular recording from a WT slice after bicuculline addition (50 μm). Within 20 min, bicuculline induced rhythmic, short epileptiform discharges (≤1.5 s in duration) that were ongoing for at least 1 h of continuous recording. Membrane potential at the beginning of recording: −60 mV. Right, CA3 intracellular recording from a 3xTg-AD slice after bicuculline. Bicuculline first induced short synchronized epileptiform discharges that were similar to those in WT slices. However, continuous perfusion with bicuculline induced prolonged epileptiform (ictal-like) discharges (>1.5 s) in 3xTg-AD slice. Membrane potential at the beginning of recording: −65 mV. ***G***, Positive correlation of intraneuronal human APP/Aβ expression in CA3 neurons and ictal-like activity in CA3 region. Correlation analyses revealed a positive relationship between intraneuronal human APP/Aβ immunoreactivity in the CA3 neurons (analyzed by 6E10, human APP/Aβ) and average duration of the five longest epileptiform discharges recorded during a 5-min period after 90 min of bicuculline application in the CA3 region of hippocampal slices from the same mice. Data from Saline-3xTg-AD (*n* = 9) and 6E10–3xTg-AD (*n* = 9) was pooled together to evaluate the correlation. The sigmoidal curve based on nonlinear regression is also shown. ***H***, ***I***, Early-onset epileptic activity in one- and two-month-old hAPP-J20 mice. Reproduced with permission from Fu et al. (2019). ***H***, Representative EEG traces from NTg and hAPP-J20 mice at one and two months of age, with epileptiform spikes at one month of age and a seizure at two months of age in hAPP-J20 mice. Electrodes were in left and right frontal cortices (LFC and RFC), hippocampus (HIP), and parietal cortex (PC). Scale bars: 1 mV, 10 s. ***I***, The number of epileptic spikes per hour in NTg or hAPP-J20 mice at one, two, and four to six months of age (*n* = 3−5 mice per genotype and age).

Several hAPP/Aβ mouse model studies have documented the presence of early-onset neuronal network hyperexcitability manifesting as epileptiform activity and seizure susceptibility, much before Aβ plaques deposition and overt cognitive impairment (Fig. 2). These studies suggest the potential role of transgenic APP and intraneuronal Aβ in neuronal network hyperexcitability before plaque deposition. Early onset of hypersynchronous activity and expression of a chronic seizures’ marker was reported in Tg2576 mice (Bezzina et al., 2015). No memory dysfunction has been reported in these mice at 1.5–2 months, and they develop Aβ plaques by 11–13 months of age (Jacobsen et al., 2006; D’Amelio et al., 2011; Stewart et al., 2011). Spontaneous epileptiform activity and an increased susceptibility to PTZ-induced seizures was observed in Tg2576 mice as early as 1.5 months of age (Fig. 2*A–C*; Bezzina et al., 2015). Additionally, higher ectopic expression of NPY in the mossy fibers was found at three months of age in these mice (Bezzina et al., 2015), suggesting that chronic seizures occur at very early stages in the course of the disease, and that their incidence likely increases with age among the Tg2576 population. Another study reported increased susceptibility to audiogenic seizures as early as three weeks of age in Tg2576 mice as compared with WT controls (Westmark et al., 2010). This early-onset audiogenic seizure susceptibility in Tg2576 mice could be suppressed by passive immunization with an anti-APP/Aβ antibody or by blockade of mGluR5 with the selective antagonist, 2-methyl-6-(phenylethynyl)pyridine hydrochloride (MPEP; Westmark et al., 2010). Additionally, a study also found increased susceptibility to PTZ-induced seizures in two-month-old Tg2576 mice (Westmark et al., 2008). Another study in Tg2576 mice, using video EEG recordings, reported synchronized, large amplitude potentials resembling interictal spikes in epilepsy at just five weeks of age, long before memory impairments or Aβ plaques deposition, suggesting epileptiform activity as a biomarker for early detection of AD (Kam et al., 2016). Also, a study in two- to four-month-old Tg2576 mice (before Aβ plaques deposition) reported increased excitability in the EC, one of the first regions to display neuropathology in AD (Duffy et al., 2015).

A study in five- to seven-month-old Tg2576 mice (still before Aβ plaques deposition) reported the presence of spike wave discharges and abnormal EEG patterns; these mice also exhibited longer durations of higher frequency brain activity, suggesting increased synchrony (Corbett et al., 2013). The Tg2576 mice with epileptiform activity exhibited increased Na_v_β2 cleavage and increased total levels of Na_v_1.1α (Corbett et al., 2013). Interestingly, the magnitude of alterations in sodium channel subunits was associated with aberrant EEG activity and impairments in the Morris water maze task (Corbett et al., 2013). As mentioned earlier, in hAPP-J20 mice, decreased levels of Na_v_1.1α, only in PV^+^ interneurons, were found which led to impaired interneuron function and aberrant neuronal activity that could be normalized by overexpressing Na_v_1.1α in interneurons (Verret et al., 2012). While Corbett and colleagues (Corbett et al., 2013) found increased rather than decreased total levels of Na_v_1.1 α in Tg2576 mice, the surface Na_v_1.1α levels were in fact reduced (Corbett et al., 2013). Overall, both studies reported a decrease in the levels of functional Nav1.1α in APP mice cortex (Verret et al., 2012; Corbett et al., 2013). Interestingly, Na_v_1.1-null mice were shown to exhibit spontaneous seizures and a significant reduction in Na^+^ currents in isolated GABAergic interneurons, but not in pyramidal cells from hippocampus, suggesting that loss of Na_v_1.1 might specifically decrease inhibitory function, thereby prompting hyperexcitability (Yu et al., 2006). Thus, Na_v_1.1 hypofunction could be a possible mechanism of neuronal network hyperexcitability in AD. A recent study also found that selective overexpression of another sodium channel subunit, Na_v_1.6, is responsible for the aberrant neuronal activity observed in hippocampal slices from three-month-old Tg2576 mice (Ciccone et al., 2019). Furthermore, the Na_v_1.6 channels were identified as a determinant of the hippocampal neuronal hyperexcitability induced by Aβ_42_ oligomers (Ciccone et al., 2019).

A study in preplaque (six- to eight-week-old) TgCRND8 mice, another hAPP/Aβ mouse model of AD, demonstrated an increased sensitivity to PTZ-induced seizures with a more severe seizure type in transgenic mice over age-matched littermate controls (Del Vecchio et al., 2004). A lower threshold and more severe seizure type in TgCRND8 mice before plaque deposition suggested that this genotype difference might be because of Aβ toxicity rather than plaque formation (Del Vecchio et al., 2004). In PS2APP mice [harboring hAPP Swedish (KM670/671NL) and PSEN2:N141I mutations; transgene expression being driven by the Thy1 and Prp promoter; overt Aβ deposition at approximately six months, with heavy plaque load in the hippocampus, frontal cortex, and subiculum at 10 months; cognitive impairment at eight months], a study employing *in vivo* recordings of LFP activity in the dentate gyrus, uncovered network hypersynchronicity as early as three months, when intracellular accumulation of Aβ (and not plaques) was observable (Fontana et al., 2017). An *in vivo* two-photon calcium imaging study in the hippocampal CA1 neurons in young (1.5-month-old) APP23xPS45 mice reported a selective increase in hyperactive neurons already before the formation of plaques, suggesting that soluble species of Aβ may underlie this impairment (Fig. 2*D*; Busche et al., 2012). Acute treatment with the γ-secretase inhibitor LY-411575 reduced soluble Aβ levels and also rescued the neuronal dysfunction (Busche et al., 2012). Furthermore, direct application of soluble Aβ could induce neuronal hyperactivity in WT mice (Busche et al., 2012). Thus, hippocampal hyperactivity was identified as a very early functional impairment in AD transgenic mice and soluble Aβ was reported to be crucial for hippocampal hyperactivity (Busche et al., 2012).

Previously, we detected the presence of early-onset neuronal network hyperexcitability at three weeks of age, much before Aβ plaque pathology and cognitive deficit, in 3xTg-AD mice (Fig. 2*E–G*; Kazim et al., 2017). The earliest cognitive deficits reported in 3xTg-AD mice are by two to three months of age (Davis et al., 2013; Stevens and Brown, 2015). However, most studies show cognitive impairment in 3xTg-AD by approximately five months of age (Oddo et al., 2003a; Billings et al., 2005). Increased susceptibility to audiogenic seizures and epileptiform discharges were observed in the hippocampal CA3 region in three-week-old 3xTg-AD mice (Fig. 2*E*,*F*; Kazim et al., 2017). In congruence with a previous study in Tg2576 mice (Westmark et al., 2010), passive immunization with an anti-APP/Aβ antibody or blockade of mGluR5 with MPEP suppressed early-onset neuronal network hyperexcitability in 3xTg-AD mice. While no amyloid plaques are present at this age in 3xTg-AD mice, they exhibit intraneuronal APP/Aβ expression; remarkably, epileptiform discharge duration positively correlated with intraneuronal transgenic hAPP/Aβ expression in the CA3 region of the hippocampus (Fig. 2*G*; Kazim et al., 2017). Another study in four- to six-month 3xTg-AD mice, an age where there is intraneuronal APP/Aβ expression but no plaques, reported the presence of synaptic hyperexcitability (Davis et al., 2014), which could be a major contributor to episodic memory deficit observed at young age in these mice (Davis et al., 2013). The familial AD mouse models which carry human APP mutation(s) and display early-onset epileptiform activity and seizure susceptibility have increased expression of intraneuronal human APP and Aβ before extracellular Aβ deposition and amyloid plaque formation (Oddo et al., 2003a,b; Billings et al., 2005; Lithner et al., 2011; Stargardt et al., 2015). In human AD brains, intraneuronal Aβ accumulation precedes plaque formation (Gyure et al., 2001; Bossers et al., 2010). In AD transgenic mice, intraneuronal Aβ deposition was described to contribute to cognitive impairment before amyloid plaque stage (Oddo et al., 2003b; Billings et al., 2005), and aberrant network excitability may be a mechanism of this cognitive deficit. Similar to Aβ, APP may also be a mediator of neuronal network hyperexcitability in AD patients and transgenic mice. Interestingly, genetic suppression of transgenic APP in a human APP mouse model of AD [tetracycline-responsive APP-transgenic mice (APP/TTA, where TTA stands for tetracycline-controlled transactivator protein)] was shown to rescue hypersynchronous network activity (Born et al., 2014). Also, individual peptides generated from APP processing may play a role in neuronal network hyperexcitability as hyperexcitability and seizure susceptibility was previously reported in mice overexpressing APP intracellular domain (AICD; Vogt et al., 2011). However, because of the intrinsic relationship of APP and Aβ in familial AD mouse models, dissecting out the differential impact of APP overexpression versus intraneuronal Aβ deposition before plaque pathology on neuronal network hyperexcitability in hAPP/Aβ mice has been experimentally daunting. Additionally, besides hAPP/Aβ, other pathogenic factors could also play a role in early-onset neuronal network hyperexcitability in AD. For instance, a study employing electrophysiological recordings in hippocampal primary neuronal cultures from embryonic (E16–E18) 3xTg-AD mice reported a causative link between the development of hyperexcitability, increased spontaneous synaptic activity, and the reactive oxygen species-dependent appearance of conglomerates of dysfunctional K_v_2.1 potassium channels (Frazzini et al., 2016).

A recent study reported epileptic spikes as early as one month of age with robust seizure activity at two months of age, before Aβ plaques deposition and overt memory dysfunction in hAPP-J20 mice (Fig. 2*H*,*I*; Fu et al., 2019). Interestingly, it was found that as early seizure activity appears, adult hippocampal neurogenesis initially increases at two months of age, however with recurrent seizure activity, a deficit in adult hippocampal neurogenesis was observed at 3, 7, and 14 months in hAPP-J20 mice (Fu et al., 2019). Adult hippocampal neurogenesis is known to play an essential role in learning and memory (Aimone et al., 2006, 2010; Sahay et al., 2011). It was proposed that the seizure activity that occurs early in disease progression in hAPP-J20 mice aberrantly stimulates neural stem cell division and accelerates depletion of the neural stem cell pool (Aimone et al., 2006, 2010; Sahay et al., 2011). At 3–3.5 months of age, when the level of neurogenesis in hAPP-J20 mice first becomes markedly reduced, deficit in a spatial discrimination memory task was found in these mice (Fu et al., 2019). This was in agreement with the previous data which showed that adult-born hippocampal neurons are critical for spatial discrimination (Sahay et al., 2011). Remarkably, chronic treatment with levetiracetam, which effectively reduces spikes and seizures in hAPP-J20 mice (Sanchez et al., 2012), normalized neurogenesis and improved spatial discrimination memory in hAPP-J20 mice, thus providing a causal link between early-onset network hyperexcitability much before Aβ plaques and cognitive deficit via recurrent epileptic activity-induced aberrant adult hippocampal neurogenesis (Fu et al., 2019). These data challenge the old concept that neuronal network hyperexcitability is a compensatory mechanism following AD-related neurodegeneration and reflect an effort of the brain that cannot keep pace with cognitive demands. In fact, the reverse seems to be true, namely that hyperexcitability is an early-onset pathologic process in AD and plays a critical role in memory dysfunction.

## The Role of tau in Neuronal Network Excitability: The Enhancement versus Suppression Conundrum

### tau, AD, and neuronal network excitability

tau is a neuronal microtubule-associated protein which plays a key role in microtubule assembly, stabilization, and axonal transport. In AD and other related tauopathies, tau is abnormally hyperphosphorylated which results in reduced binding of tau to microtubules, and subsequent accumulation as NFTs, leading to neurodegeneration and cognitive impairment (Grundke-Iqbal et al., 1986a,b; Iqbal et al., 2016). tau pathology is known to be better correlated with cognitive deficit in AD than Aβ pathology (Nelson et al., 2012), and tau spread from EC to other cortical areas via connected neuroanatomical circuitry is a critical process in the progression of AD (de Calignon et al., 2012; Liu et al., 2012). Several studies have looked into the role of tau in neuronal network hyperexcitability in AD (Roberson et al., 2007; García-Cabrero et al., 2013; Holth et al., 2013; Angulo et al., 2017; Hatch et al., 2017; Mondragón-Rodríguez et al., 2018b; Busche et al., 2019); however, the data are conflicting with the precise role remaining yet to be elucidated. Table 2 summarizes the studies evaluating neuronal network excitability in tau-based mouse models of AD.

**Table 2:** Summary of studies analyzing neuronal network excitability in tau mouse models

Author(s) and publication year	Mouse model/transgene(s)	Age/stage of pathology	Neuronal network excitability status	Experimental paradigm/neuronal network excitability observation(s)
Rocher et al. (2010)	rTg4510 (htau P301L)	8.5 months, NFTs and neurodegeneration	Increased	Experimental paradigm: *in vitro* whole cell patch clamp recordings of layer 3 frontal cortex pyramidal neurons. Findings: increased action potential firing rates and a significantly depolarized resting membrane potential in transgenic mice slices, independent of NFTs.
Hoover et al. (2010)	rTg4510 (htau P301L) Rat hippocampal neurons transfected with hτP301L	rTg4510 cultured hippocampal neurons from E18: DIV 22–30, decreased excitatory glutamate receptor levels. Rat hippocampal neurons transfected with hτP301L: DIV 22–30, increased phospho-tau	Decreased	Experimental paradigm: *in vitro* hippocampal neurons electrophysiology, mEPSCs recording. Findings: reduced mEPSCs frequency and amplitude both in rTg4510 cultured hippocampal neurons and rat hippocampal cultured neurons transfected with hτP301L.
Crimins et al. (2011)	rTg4510 (htau P301L)	9 months, NFTs and neurodegeneration	Increased	Experimental paradigm: *in vitro* whole cell patch clamp recordings of layer 3 frontal cortex pyramidal neurons. Findings: increased spontaneous synaptic activity (increased frequency of sEPSCs).
Crimins et al. (2012)	rTg4510 (htau P301L)	< 4 (1–3) mo and > 8 (9–13) mo; soluble hyperphosphorylated tau species at <4 months, NFTs and neurodegeneration at >8 months	Increased	Experimental paradigm: *in vitro* whole cell patch clamp recordings of layer 3 frontal cortex pyramidal neurons. Findings: increased excitability both in early and advanced tauopathy. Depolarized resting membrane potential, an increased depolarizing sag potential and increased action potential firing rates—all indicative of hyperexcitability. Hyperexcitability reversed by suppression of human mutant tau transgene.
Menkes-Caspi et al. (2015)	rTg4510 (htau P301L)	3 months, accumulation of hyperphosphorylated and misfolded tau in cortex; 5 months, pathologic tau and NFTs in cortex	Decreased	Experimental paradigm: *in vivo* intracellular recordings from frontal cortex in anesthetized mice, *In vivo* extracellular recordings/LFPs in awake behaving mice. Findings: reduced activity both of single neocortical pyramidal cells and of the neocortical network including decreased firing rates and altered firing patterns.
Witton et al. (2016)	rTg4510 (htau P301L)	7–8 months, NFTs and neurodegeneration	Increased	Experimental paradigm: *in vivo* hippocampal CA1 electrophysiology recordings, both single-unit and LFPs. Findings: increased propensity of excitatory pyramidal neurons in hippocampus to fire action potentials in a phase locked manner during SWRs; inhibitory interneurons were less likely to fire phase‐locked spikes during SWRs.
Hatch et al. (2017)	rTg4510 (htau P301L, 13-fold higher human tau expression as compared with endogenous tau) pR5 (htau P301L, at lower level than rTg4510, 0.7-fold higher human tau as compared with endogenous tau)	rTg4510: 1–2 months, early stage tauopathy before overt tau hyperphosphorylation and synaptic impairment 4–6 months, mid-stage with extensive tau hyperphosphorylation and impairment of synaptic activity and spatial memory 12–14 months, late stage with synaptic loss and neurodegeneration PR5: 15–17 months, tau pathology in hippocampus	Decreased	Experimental paradigm: *in vitro* whole cell patch clamp recordings from hippocampal CA1 pyramidal neurons. Findings: reduced action potential firing rate because of a depolarization shift in action potential generation and reduced action potential amplitude at all ages in the CA1 pyramidal neurons of P301L mice. pR5 mice CA1 pyramidal neurons showed less severe action potential impairment compared with rTg4510, including action potential depolarization shift and reduced action potential amplitude.
Busche et al. (2019)	rTg4510 (htau P301L) rTg21221 (htau overexpression)	rTg4510: 6–12 months, tau aggregation and NFTs 3–4 months; soluble tau. rTg21221: 6–12 months, human tau overexpression.	Decreased	Experimental paradigm: *in vivo* two-photon Ca^2+^ imaging of neurons in layer 2/3 of the cortex. Findings: strong reduction in cortical activity as reflected by increased number of silent neurons in both 6- to 12- and 3- to 4-month-old rTg4510 mice. Reduction in cortical activity in 6- to 12-month-old rTg2210 mice. Reducing tau in 3- to 4-month-old rTg4510 mice decreased the number of silent neurons.
Van Erum et al. (2020)	tau58/4 (htau P301S)	3 months, htau and phospho-tau in the frontal cortex and pons 12–15 months, NFTs throughout the brain.	Increased	Experimental paradigm: video EEG recordings; PTZ-induced seizure susceptibility. Findings: increased PTZ-induced seizure susceptibility in young (3-month-old) tau58/4 mice as compared with age-matched WT littermates. Young tau58/4 animals displayed more severe seizures and had a reduced latency to the first seizure compared with WTs. While, age-related differences in susceptibility could be demonstrated for both genotypes, old tau58/4 did not exhibit a significantly higher seizure susceptibility as compared with WT mice.
Marinković et al. (2019)	P301S (htau P301S)	2 m, injected with tau preformed fibrils for NFTs seeding, evaluated for cortical activity up to 50 d after injections	Decreased	Experimental paradigm: *in vivo* two-photon Ca^2+^ imaging of neurons in layer 2/3 of the cortex in awake, head-fixed mice. Findings: strong reduction in cortical activity, independent of NFTs presence, suggesting the impairing role of soluble, mutated tau protein species.
Mondragón-Rodríguez et al. (2018b)	3xTg-AD (htau P301L, hAPP Swedish, hPSEN1)	1 month, increased phospho-tau, intraneuronal APP/Aβ, prior to cognitive impairment.	Decreased	Experimental paradigm: *in vitro* whole cell patch clamp recordings from hippocampal CA1 pyramidal neurons; *in vitro* LFPs recordings from hippocampal slices. Findings: no difference in amplitude and frequency of action potentials between 3×Tg-AD and non-Tg CA1 pyramidal neurons. Overall, the young 3×Tg-AD mice showed less excitable hippocampal network activity, likely related to abnormally hyperphosphorylated tau at microtubule domain region (MDr).
Ahnaou et al. (2017)	Tg tau P301L (h tau P301L)	3 months, injected with preformed tau fibrils to induce tau aggregation.	Normal	Experimental paradigm: *in vivo* EEG recordings. Network oscillations, phase amplitude cross frequency coupling, mismatch negativity (MMN) of event related brain potentials, and coherence was analyzed. Findings: weakening of θ oscillations, drastic impairments in θ–γ oscillations phase-amplitude cross frequency coupling, and disrupted MMN complex amplitude (all vital for memory and learning performance) induced by tau seeding. No epileptiform activity or network hyperexcitability.
Angulo et al. (2017)	EC-htau (hτP301L) EC-hAPP (hAPP) EC-hAPP/htau (hAPP, hτP301L)	EC-htau: 2.5–3.5 months, htau and phospho-tau accumulation in EC. EC-hAPP: 2.5–3.5 months, soluble Aβ.	Normal/ resistance to induced-hyperexcitability in EC-htau mice	Experimental paradigm: *in vitro* hippocampal EC/subiculum electrophysiology recordings: single electrode evoked and sEFPs, single neuron patch clamp, and extracellular multielectrode recordings. Findings: mutated htau induced resistance to EC-hippocampus hyperexcitability in EC-htau mice evidenced by resistance to increased network activity evaluated by sEFP durations after GABA_A_ blockade with picrotoxin. Increased neuronal excitability in EC in EC-hAPP mice evidenced by higher frequency of relatively prolonged sEFPs in lateral EC and epileptiform-ictal like discharges in medial EC. While no differences were observed in sEFPs duration and frequency in EC/CA1/subiculum regions (using multielectrode recordings) between EC-hAPP/htau mice and WT mice, a smaller percentage of slices displayed epileptiform discharges. Co-expression of hAPP and htau produced an intermediate phenotype, mostly driven by tau
Maeda et al. (2016)	htau-A152T	4–9 months, soluble tau	Increased	Experimental paradigm: *in vivo* EEG recordings in awake behaving mice; epileptic spikes quantification both at baseline and after injection of non-convulsive dose of PTZ. Findings: increased epileptic spike counts both at baseline and after PTZ injection in htau-A152T mice as compared with non-Tg controls.
Decker et al. (2016)	htau-A152T	12–14 months, accumulation of hyperphosphorylated and missorted tau, neurodegeneration, and synaptic loss in hippocampal CA3 region. Increased phospho-tau in hippocampal slice cultures at DIV 10.	Increased	Experimental paradigm: *in vivo* EEG recordings in awake behaving mice; epileptic spikes quantification both at baseline and after injection of non-convulsive dose of PTZ. Findings: enhanced basal synaptic transmission in CA3 region of the hippocampus in htau-A152T mice (increased fEPSPs in mossy fiber pathway in acute slices from 12-month-old mice and increased somatic field potentials in stratum pyramidale of area CA3 in organotypic hippocampal slices at DIV 30. Increase in picrotoxin-induced epileptiform burst frequency as well as in firings per burst in organotypic slice cultures expression htau-A152T mutation (both at DIV 10 and 30) which was prevented by ceftriaxone (stimulates astrocytic glutamate uptake via the transporter EAAT2/GLT1).
Das et al. (2018)	htau-A152T	4–6 months; soluble tau	Increased	Experimental paradigm: *in vivo* EEG recordings; epileptic spikes quantification. Findings: increased epileptic spike counts at resting state in htau-A152T mice as compared with non-Tg controls. Antiepileptic drug levetiracetam treatment reduced epileptic spike counts in htau-A152T mice.
García-Cabrero et al. (2013)	FTDP-17 mice (htau G272V, P301L, and R406W; Overexpression of human tau isoform with 2 N-terminal inserts, 4-microtubule-binding-repeat elements)	1–5, 6–14, and 15–22 months; mutant tau transgene overexpressed at 3 m of age, activated microglia at 4 months, reactive astrocytes at 9 months, and phospho-tau aggregates at 18–20 months	Increased	Experimental paradigm: *in vivo* video EEG recordings and PTZ-seizure susceptibility testing. Findings: spontaneous epileptiform activity and epileptic seizures in 70% of FTDP-17 mice at the age of 5.5 m and thereafter. Increased PTZ-induced seizures susceptibility at 6–14 months of age and thereafter.

Aβ, amyloid β; EC, entorhinal cortex; DIV, days *in vitro*; EAAT2, excitatory amino acid transporter 2; EEG, electroencephalogram; fEPSPs, field EPSPs; FTDP, frontotemporal dementia with parkinsonism; GLT1, glutamate transporter 1; hAPP, human amyloid β precursor protein; hPSEN, human presenilin; LFPs, local field potentials; mEPSCs, miniature EPSCs; NFTs, neurofibrillary tangles; PTZ, phenylenetetrazole; sEFPs, spontaneous extracellular field potentials; sEPSCs, spontaneous EPSCs; SWDs, spike-wave discharges; SWRs, sharp-wave ripples; Tg, transgenic; WT, wild type.

### Tau promotes neuronal network hyperexcitability both in early stages before NFTs and at advanced stages of tau pathology

#### tau and epilepsy

tau has classically been considered to promote neuronal network hyperexcitability and to have an enabling role for epileptogenesis (Vossel et al., 2017; Sánchez et al., 2018). Hyperphosphorylated tau deposits were reported in epilepsy patients’ brains (Thom et al., 2011; Tai et al., 2016). Additionally, tau hyperphosphorylation was also demonstrated in experimental rodent models of epilepsy (Crespo-Biel et al., 2007; Tian et al., 2010). Interestingly, hyperphosphorylated tau in patients with refractory TLE was reported to correlate with accelerated cognitive decline (Tai et al., 2016). A recent study in clinically normal older adults using tau PET scan found that temporal lobe tau accumulation was associated with hippocampal hyperactivity (demonstrated by increased fMRI activity; Huijbers et al., 2019). Similarly, another recent study using task-related fMRI in combination with measures of tau pathology in CSF reported that higher CSF tau levels were related to hippocampal hyperactivity and object mnemonic discrimination in older adults (Berron et al., 2019). Also, accelerated kindling epileptogenesis was observed in rTg4510 mutant human tau mice (with 13-fold tau overexpression) but not in tau knock-out mice (Liu et al., 2017). Furthermore, genetic deletion of tau was also shown to attenuate neuronal network hyperexcitability in mouse and *Drosophila* models of hyperexcitability (Holth et al., 2013). Also, genetic reduction of tau in a mouse model of Dravet syndrome, a severe childhood epilepsy caused by mutations in the human SCN1A gene encoding the voltage-gated sodium channel subunit Na_v_1.1, was reported to reduce the frequency of spontaneous and febrile seizures and premature mortality, to decrease epileptic interictal spikes *in vivo* and drug-induced epileptic activity in brain slices *ex vivo*, and to ameliorate learning and memory deficits (Gheyara et al., 2014). These data further suggested that cognitive deficit could be directly or indirectly linked to tau-dependent epileptic activity. Taken together these data hint toward a proepileptic role of tau. Recent studies have also shown, in turn, that network hyperexcitability enhances tau propagation and tau pathology (Pooler et al., 2013; Wu et al., 2016). Accordingly, modeling chronic TLE in 3xTg-AD mice (harboring mutant human APP, presenilin and tau proteins) was reported to enhance tau phosphorylation in the temporal lobe structures (Yan et al., 2012). This may potentially be a vicious cycle where early tau deposition enhances neuronal network excitability which in turn further increases tau release and propagation.

#### High-frequency oscillations or SWRs, epilepsy, and tau

High-frequency neuronal oscillations (HFOs; 100–250 Hz) in the hippocampus, known as SWRs, synchronize the firing behavior of groups of neurons and are thought to play an important role in driving Hebbian synaptic plasticity and memory consolidation (Buzsáki et al., 1992; Sadowski et al., 2011). Hippocampal SWRs drive the synchronous co-activation of local populations of pyramidal neurons and interneurons (Csicsvari et al., 1999; Klausberger and Somogyi, 2008). Hippocampal SWRs were suggested to be a cognitive biomarker for episodic memory and planning. High-frequency SWRs with spectral frequencies in the range of 250–600 Hz, called fast ripples, have been described in the brains of epileptic patients and rodents (Worrell et al., 2004; Bragin et al., 2010). In animal models of TLE, fast ripples occur in the dentate gyrus, CA1, and CA3 areas of hippocampus, subiculum, and EC in rats that exhibit recurrent spontaneous seizures (Bragin et al., 1999). A recent study reported that early hippocampal SWR abnormality predicts later learning and memory impairments in an AD mouse model (Jones et al., 2019).

Previously, disrupted hippocampal SWR associated spike dynamics (frequency and temporal structures) were reported in a tau-based transgenic mouse model of dementia, i.e., rTg4510 transgenic mice that express aggregating human tau P301L [a frontotemporal lobe dementia (FTD) mutation] and display NFTs but no Aβ pathology (Witton et al., 2016). On *in vivo* electrophysiological recordings in the hippocampus of seven- to eight-month-old rTg4510 mice, an age when NFTs are rampant and neurodegeneration is well established in these mice (Ramsden et al., 2005), it was found that excitatory pyramidal neurons were more likely to fire action potentials in a phase locked manner during SWRs; conversely, inhibitory interneurons were less likely to fire phase locked spikes during SWRs (Witton et al., 2016). These data indicated a reduced inhibitory control of hippocampal network events and pointed toward a hyperexcitability-based mechanism which may underlie the cognitive impairments in this model of dementia (Witton et al., 2016).

#### Tau reduction decreases neuronal network hyperexcitability in Aβ mice and reduces seizure susceptibility in WT mice

Table 3 summarizes the studies evaluating the effect of tau reduction on neuronal network excitability in Aβ and WT mice. Previously, genetic reduction of endogenous tau was reported to reduce interictal spiking and spontaneous seizures besides ameliorating cognitive deficit without affecting Aβ pathology in the J20 (hAPP) mouse model of AD (Roberson et al., 2007). In fact, tau reduction also slowed the onset of PTZ-induced seizures and lowered the susceptibility to kainate-induced seizures in WT mice without hAPP (Roberson et al., 2007), suggesting a role for endogenous tau in enhancing neuronal network excitability. Another study in aged tau knock-out mice, further corroborated decreased PTZ-induced seizure susceptibility (Li et al., 2014).

**Table 3:** Summary of studies analyzing the effect of tau reduction on network excitability in Aβ mice and in WT mice

Author(s) and publication year	Mouse model/ transgene(s)	Age/stage of pathology	Baseline neuronal network excitability status	Experimental paradigm and tau reduction strategy/effect of tau reduction on neuronal network excitability
Roberson et al. (2007)	hAPPJ20/tau^+/+^, hAPPJ20/tau+/−, hAPPJ20/tau*^−/−^*, tau^+/+^, tau+/−, tau*^−/−^*.hAPP(J20): hAPP_Swe_, hAPP_Ind_, PDFG promoter	hAPP/tau^+/+^: 4–7 months, Aβ plaques, neurodegeneration	Increased in hAPP/tau^+/+^	Experimental paradigm: *in vivo* PTZ-induced and kainate-induced seizure susceptibility evaluation. tau reduction achieved through crossing hAPPJ20 line with tau knock-out (tau*^−/−^*). Findings: tau reduction increased resistance to both PTZ-and kainate-induced seizures. Seizures were less severe in hAPP/*tau*^+/–^ and hAPP/*tau*^–/–^ mice than in hAPP/*tau*^+/+^ mice. Seizures were also less severe in *tau*^–/–^ mice than in *tau*^+/+^ mice. The onset of seizure was also delayed by tau reduction. tau reduction also ameliorated learning and memory deficits in hAPPJ20 mice.
Roberson et al. (2011)	hAPPJ20/tau^+/+^, hAPPJ20/tau+/−, hAPPJ20/tau*^−/−^*, hAPPJ9/tau^+/+^, hAPPJ9/tau*^−/−^* tau^+/+^, tau*^−/−^*.	7–14 months for *in vivo* EEG detection of frequency of epileptiform spikes: Aβ plaques, neurodegeneration. 5–8 months for *in vivo* PTZ-induced seizure susceptibility evaluation: Aβ plaques, neurodegeneration	Increased in hAPPJ20/tau^+/+^ and hAPPJ9/tau^+/+^ mice	Experimental paradigm: *in vivo* PTZ-induced seizure susceptibility evaluation. *In vivo* EEG detection of frequency of epileptiform spikes in freely moving mice. *In vitro* epileptiform discharges in area CA1 of the hippocampus after bicuculline administration in acute slices. tau reduction achieved via crossing hAPPJ20 or hAPPJ9 line with tau knock-out (tau*^−/−^*) mice. Findings: tau reduction decreased PTZ-induced seizure severity and frequency of generalized epileptiform spikes in hAPPJ20 mice. tau reduction also prevented bicuculline-induced epileptiform bursting in acute hippocampal slices from WT (tau*^−/−^*) and hAPPJ20 mice.
DeVos et al. (2013)	WT (C57BL/6J), tau*^−/−^*	3–5 months, no pathology	Normal	Experimental paradigm: *in vivo* EEG recordings, baseline and after picrotoxin administered via reverse microdialysis. *In vivo* PTZ-induced seizure susceptibility evaluation. tau reduction was achieved via ASOs. Findings: reduction in normalized spike frequency after picrotoxin administration in ASO-treated WT mice and tau*^−/−^* as compared with controls. Total tau protein levels in the hippocampus of mice highly correlated with normalized spike frequency. PTZ-induced seizure severity was significantly reduced in ASO-treated WT mice. Seizure severity and tau protein levels correlated well in all tested mice.
Li et al. (2014)	tau^+/+^, tau+/−, tau*^−/−^*. (all on C57Bl/6J background)	24 months, no pathology, age-appropriate cognitive function	Normal	Experimental paradigm: *in vivo* PTZ-induced seizure susceptibility evaluation. tau reduction achieved via genetic homozygous or heterozygous knock-out. Findings: PTZ-induced seizure severity was significantly reduced in tau knock-out aged mice. Also, aged tau*^+/−^* and tau*^−/−^* mice had longer seizure latencies than tau*^+/+^* mice.
Ittner et al. (2010)	APP23 (APP_Swe_, Thy1 promoter), Δtau74 **(**amino acids 256–441 removed from the longest human tau isoform, htau40), tau*^−/−^*, Δtau74.tau*^−/−^*, APP23. Δtau74, APP23. tau*^−/−^*, APP23. Δtau74.tau*^−/−^*	APP23: 2–3 months, no plaques. Δtau74: 2–3 months, tau missorting, normal endogenous tau but negligible phospho-tau	Increased in APP23 mice	Experimental paradigm: *in vivo* PTZ-induced seizure susceptibility evaluation. tau reduction achieved via crossing APP23 line with Δtau74 or tau*^−/−^* mice. Findings: seizure severity was significantly reduced in Δtau74, tau*^−/−^*, and Δtau74. tau*^−/−^* compared with the WT, while the latency to develop severe convulsion was increased. APP23 mice presented with a reduced convulsion latency and showed the most severe seizure response. However, when APP expression was combined with Δtau expression or tau deficiency, this significantly decreased seizure severity, reduced fatality, and increased convulsion latency. The double mutant Δtau74.tau*^−/−^* prevented severe seizures better than Δtau74 or tau*^−/−^*alone, on both WT and APP23 backgrounds.

Aβ, amyloid β; ASOs, antisense oligonucleotides; EEG, electroencephalogram; hAPP, human amyloid β precursor protein; PDGF, platelet-derived growth factor; PTZ, phenylenetetrazole; WT, wild type.

Antisense oligonucleotides-mediated reduction of endogenous tau throughout the entire CNS (brain and spinal cord tissue, interstitial fluid, and CSF) of WT mice was found to reduce seizure susceptibility in two chemically-induced seizure models; mice with reduced tau protein had less severe seizures than control mice (DeVos et al., 2013). In fact, total tau protein levels and seizure severity were highly correlated, such that those mice with the most severe seizures also had the highest levels of tau (DeVos et al., 2013). This finding was more important given the fact that in this study tau reduction was achieved in adult mice through antisense oligonucleotides as compared with genetic ablation of tau where a developmental compensation may account for the protection against seizures (DeVos et al., 2013). It was also later shown that the antisense oligonucleotide-mediated tau reduction prevented hippocampal volume loss and neuronal death, extended mouse survival, and reduced pathologic tau seeding in P301S mouse model of tauopathies (DeVos et al., 2017).

Further confirming the role of tau in mediating Aβ-induced hyperexcitability, another study found that tau reduction prevented spontaneous epileptiform activity in multiple lines of hAPP mice (Roberson et al., 2011). tau reduction was also found to reduce the severity of spontaneous and chemically-induced seizures in mice overexpressing Aβ (Roberson et al., 2011). Additionally, whole-cell current recordings from acute hippocampal slices of hAPP mice with tau exhibited increased spontaneous and evoked excitatory currents, reduced inhibitory currents, and NMDAR dysfunction (Roberson et al., 2011). tau reduction increased inhibitory currents and normalized excitation/inhibition (E/I) balance and NMDAR-mediated currents in hAPP mice (Roberson et al., 2011).

tau protein was also reported to be mediating Aβ-induced axonal transport deficits and synaptic long-term potentiation (LTP) alterations in hAPP mice, both of which were rescued by tau knock-out (Vossel et al., 2010; Shipton et al., 2011). tau-dependent depletion of K_v_4.2 (a dendritic potassium channel important for regulating dendritic excitability and synaptic plasticity) and dendritic hyperexcitability in the CA1 region of the hippocampus were also observed in an AD mouse model overexpressing Aβ (Hall et al., 2015). Additionally, a dendritic function of tau in Aβ-dependent excitotoxicity via postsynaptic targeting of the Src kinase Fyn, a substrate of which is the NMDAR, was previously shown (Ittner et al., 2010). Missorting of tau in transgenic mice expressing truncated tau (Δtau) and absence of tau in tau^−/−^ mice were both found to disrupt postsynaptic targeting of Fyn, and reduce PTZ-induced seizure susceptibility in mice (Ittner et al., 2010). Notably, in APP23 mice, when APP expression was combined with Δtau expression or tau deficiency, this significantly decreased seizure severity, reduced fatality, and increased convulsion latency (Ittner et al., 2010). An NMDAR/PSD-95/tau/Fyn complex was shown to mediate Aβ-dependent neuronal network hyperexcitability (Ittner et al., 2010, 2016; Ittner and Götz, 2011). Overall, these data demonstrated that tau protein is an important mediator of Aβ-induced neuronal network hyperexcitability.

Expression of ApoE4, an AD risk factor allele, is associated with neuronal network hyperexcitability in mice (Hunter et al., 2012). ApoE4 mice were shown to exhibit GABAergic inhibitory interneuron loss accompanied by cognitive impairments associated with abnormally hyperphosphorylated tau, and this pheontype was rescued by tau reduction (Li et al., 2009; Andrews-Zwilling et al., 2010). These data further suggest a role for tau in ApoE4 mediated neuronal network hyperexcitability in AD.

#### Neuronal network hyperexcitability in mouse models of tauopathies

**Figure 3.**
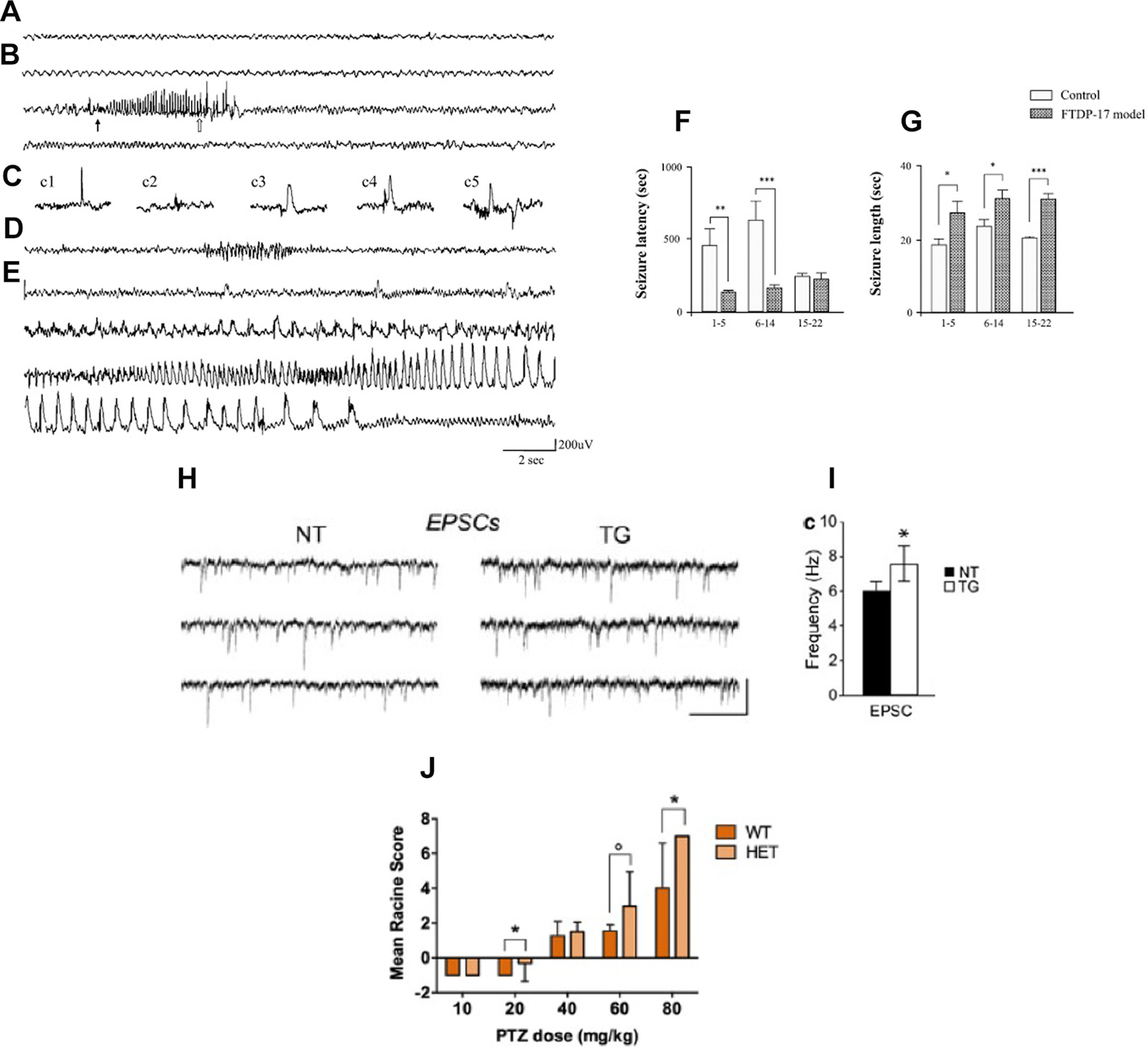
Neuronal network hyperexcitability in tau mouse models. ***A–G***, Hyperexcitability and epileptic seizures in a mouse model of a tauopathy (FTDP-17). Reproduced from García-Cabrero et al. (2013) with permission from Elsevier. ***A***, Intracranial recording of background activity (6–7 Hz) in control mice. ***B***, Spindle-shaped polyspike discharge at 8–10 Hz, during an initial tonic phase (thin arrow indicates the beginning) and a short clonic phase (open arrow signals the beginning) in a FTDP-17 mouse. ***C***, Spontaneous interictal epileptic activity in FTDP-17 mice corresponding to (c1) single spike, (c2) polyspike, (c3) slow wave, and (c4, c5) polyspike-wave discharges. ***D***, Nonconvulsive spontaneous seizure with EEG correlates corresponding to rhythmic, spindle-shaped discharges. ***E***, Spontaneous generalized tonic–clonic seizure in a FTDP-17 mouse manifested in the EEG record as generalized low-frequency (3–6 Hz) poyspike-wave discharge, 36 s in length. Figure shows the records from monopolar electrodes placed over the left frontal cortex with the reference electrodes implanted posterior to λ. ***F***, ***G***, Analysis of seizure latency and length of PTZ-induced generalized seizures in FTDP-17 mice. Mice at three different age spans (1–5, 6–14, and 15–22 months) were injected with a convulsive dose of PTZ (50 mg/kg). ***F***, The time interval between drug administration and development of generalized tonic–clonic seizures and (***G***) the seizure length were measured. Data are presented as mean ± SEM. Student’s *t* test was performed for statistical evaluation; *p <* *0.05, **p <* *0.01, ***p <* *0.001 (*n* = 15–24). ***H***, ***I***, Increased spontaneous synaptic activity in whole-cell patch clamp recordings of layer 3 frontal cortex pyramidal neurons of rTg4510 (hTau P301L) mice. Reproduced with permission from Crimins et al. (2011). Increased frequency of sEPSCs in TG (rTg4510) cells. ***H***, Representative sEPSCs from non-transgenic (NT) and TG cells. ***I***, Bar graphs of mean frequency sEPSCs in NT and TG cells. ***J***, Increased PTZ-induced seizure susceptibility in three-month-old Tau58/4 (htau P301S) mice. Young HET (Tau58/4) mice had higher mean severity scores than WT littermates. Reproduced with permission from Van Erum et al. (2020).

Besides AD, abnormal forms of hyperphosphorylated tau also accumulate in other tauopathies such as FTD, corticobasal degeneration (CBD), and progressive supranuclear palsy (PSP). The tauopathy mouse models, particularly those carrying FTD mutations, provide a unique opportunity to study AD-like tau pathology (Goedert et al., 2012). Autosomal dominant FTD with parkinsonism linked to chromosome 17 (FTDP-17) is a tauopathy characterized by the presence of abnormally hyperphosphorylated tau deposits in the absence of Aβ pathology (Foster et al., 1997). Previously, hyperexcitability and epileptic seizures were reported in FTDP-17 mouse model, a transgenic mouse line over-expressing a human tau isoform with 2 N-terminal inserts, 4-microtubule-binding-repeat elements and with the three FTDP-17-linked mutations G272V, P301L, and R406W (Fig. 3*A–G*; García-Cabrero et al., 2013). Mutant tau transgene was profusely expressed as early as three months of age in these mice, two months before the appearance of spontaneous epileptic activity (García-Cabrero et al., 2013). Thus, human FTDP-17 mutant tau expression in the absence of Aβ pathology in this model was sufficient to lead to physiological dysfunction which resulted in the epileptic activity and seizures (García-Cabrero et al., 2013). Recently, another FTD-causing tau mutation (V337M) was reported to impair activity-dependent plasticity of the cytoskeleton in the axon initial segment (AIS), and extracellular recordings by multielectrode arrays (MEAs) revealed that the V337M tau mutation in human neurons led to an abnormal increase in neuronal activity in response to chronic depolarization (Sohn et al., 2019).

Previously, employing *in vitro* intracellular recordings, neuronal network hyperexcitability was reported in rTg4510 human tau transgenic mice (Rocher et al., 2010; Crimins et al., 2011, 2012). It was reported, using whole cell patch clamp recordings, that mutated tau led to increased action potential firing rate in layer three frontal cortical pyramidal neurons in 8.5-month-old rTg4510 mice slices, independent of NFTs formation (Rocher et al., 2010). Another study further corroborated this data, demonstrating increased frequency of spontaneous EPSCs (sEPSCs) in layer three pyramidal neurons of frontal cortical slices of nine-month-old rTg4510 mice, and proposing increased excitability as a compensatory homeostatic response by surviving neurons to neurodegeneration in tauopathies (Fig. 3*H*,*I*; Crimins et al., 2011). Interestingly, a subsequent study by the same group found neuronal hyperexcitability in layer three pyramidal neurons of frontal cortical slices in both early (less than four months of age) and late (more than eight months of age) stages of tauopathy in rTg4510 mice (Crimins et al., 2012). The measures of hyperexcitability found in layer three cortical neurons at both early and late stages of tauopathy in rTg4510 mice included depolarized resting membrane potential, an increased depolarizing sag potential and increased action potential firing rate (Crimins et al., 2012)

A previous study found that the A152T-variant of human tau (htau-A152T), known to increase the risk for both AD and non-AD tauopathies (Coppola et al., 2012), when expressed in transgenic mice neurons led to not only age-dependent cognitive decline, neurodegeneration, and gliosis, but also caused intermittent epileptic spike activity detectable by EEG (Maeda et al., 2016). The epileptic spikes were more abundant in htau-A152T mice and less abundant in htau-WT mice (carrying human tau overexpression) as compared with non-transgenic controls (Maeda et al., 2016). An earlier *in vitro* study employing acute hippocampal slice and hippocampal slice cultures found enhanced basal synaptic transmission and an increase in picrotoxin-induced epileptiform burst frequency as well as in action potential firing per burst in the CA3 region of the hippocampus in htau-A152T mice (Decker et al., 2016). Increased extracellular glutamate was also observed in hippocampal slice cultures form htau-A152T mice; the increased picrotoxin-induced epileptiform activity in htau-A152T slice cultures was prevented by ceftriaxone which stimulates astrocytic glutamate uptake via the transporter EAAT2/GLT1 (Decker et al., 2016). In another study, the htau-A152T expression in mice brain was also found to increase the power of brain oscillations in the 0.5- to 6-Hz range (δ-θ) more than the increase induced by only htau expression when compared with non-transgenic controls (Das et al., 2018). These data suggest the possibility that tau-mediated neuronal network hyperexcitability may not only be dependent on its expression level but also on its sequence. Remarkably, genetic ablation of endogenous tau in Mapt^−/−^ mice reduced the power of these brain oscillations when compared with WT controls (Das et al., 2018). Additionally, suppression of htau-A152T production in doxycycline-regulatable transgenic mice reversed their abnormal network activity (Das et al., 2018). Also, treatment of htau-A152T mice with the antiepileptic drug levetiracetam persistently reversed their brain dysrhythmia and network hypersynchronization (Das et al., 2018).

A recent study examined PTZ-induced seizure susceptibility in tau58/4 mice expressing the human 4R/0N tau isoform that contains the point mutation of proline-to-serine in codon 301 (P301S) of the *MAPT* gene (Fig. 3*J*; Van Erum et al., 2020). Overexpression of human tau is present in these mice from birth in heterozygous animals. In the cerebrum, hyperphosphorylated tau arises at the level of the pons and frontal cortices at three months of age. NFTs formation is observed at the age of six months, and at 12 months; NFTs are diffusely present in the frontal cortex and the pons and also appear in the cerebellum, midbrain, and parietal cerebral cortex (Van Erum et al., 2020). The study found an increased PTZ-induced seizure susceptibility in young (three months old; Fig. 3*J*), but not in old (12- to 15-month-old) tau58/4 mice. Young tau58/4 animals displayed more severe seizures and had a reduced latency to the first seizure compared with WT littermates (Van Erum et al., 2020). Also, age-related differences in susceptibility could be demonstrated for both genotypes (Van Erum et al., 2020).

### Tau suppresses neuronal network excitability even before development of NFTs: evidence for the counter argument

#### *In vivo* and *in vitro* electrophysiological evidence of tau-mediated suppression of neuronal activity in mouse models of tauopathies

**Figure 4.**
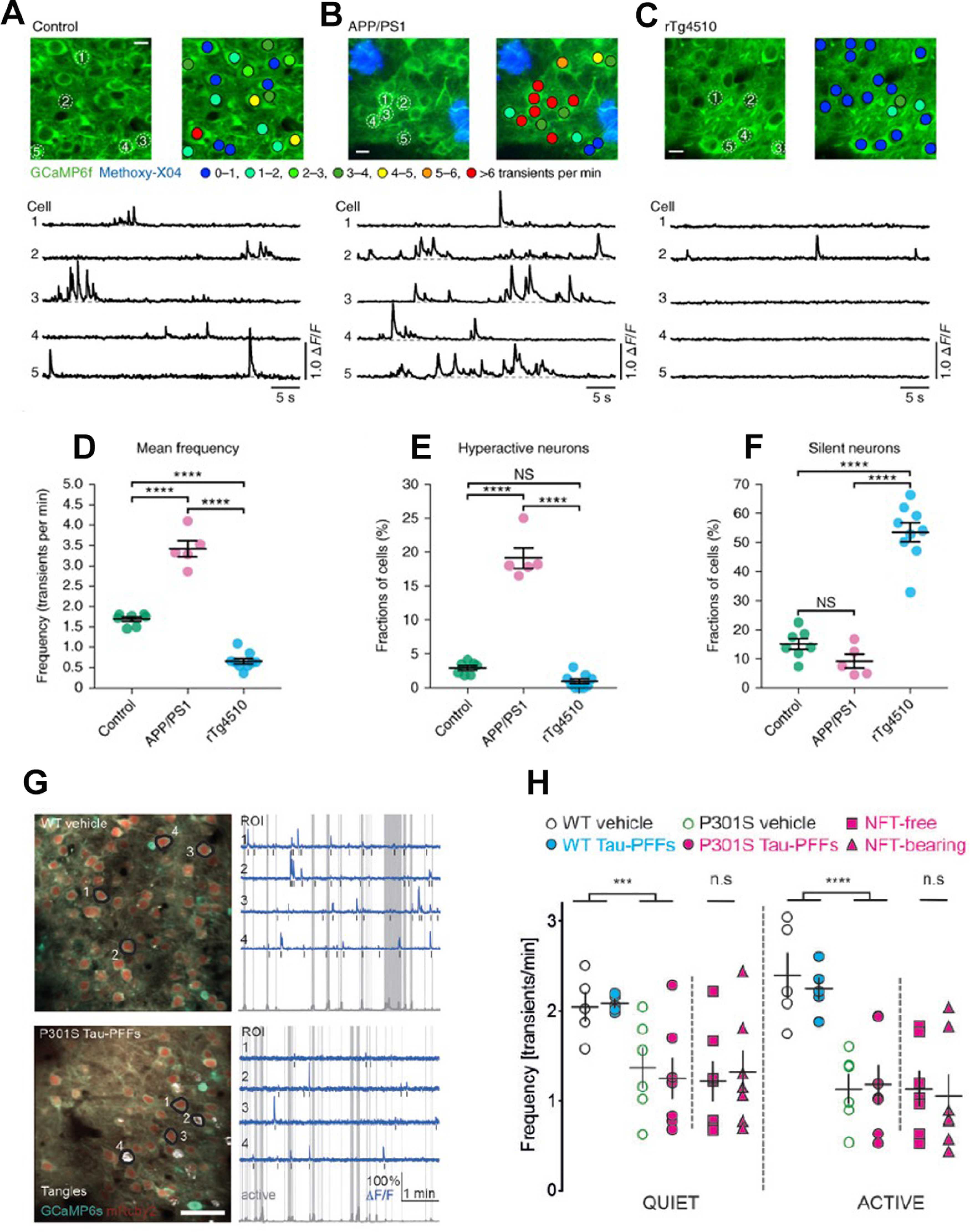
*In vivo* evidence of suppression of neuronal activity in tau mouse models. ***A–F***, Neuronal silencing in rTg4510 mice as compared with neuronal hyperactivity in APP/PS1 mice. Reproduced with permission from Busche et al. (2019). ***A–C***, top, *In vivo* two-photon fluorescence images of GCaMP6f-expressing (green) layer 2/3 neurons in the parietal cortex and corresponding activity maps from WT controls (***A***), APP/PS1 (***B***), and rTg4510 (***C***) mice. In APP/PS1 mice, plaques were labeled with methoxy-X04 (blue); in the activity maps, neurons were color-coded as a function of their mean Ca^2+^ transient activity. Scale bars: 10 μm. Bottom, spontaneous Ca^2+^ transients of neurons indicated in the top panel. ***D***, Mean neuronal frequencies for controls (1.69 ± 0.05 transients per minute), APP/PS1 (3.42 ± 0.20 transients per minute), and rTg4510 (0.66 ± 0.07 transients per minute); *F*_(2,18)_ = 171.2, *p* = 1.93 × 10^−12^. All *post hoc* multiple comparisons between genotypes were highly significant: *p* = 5.42 × 10^−9^ for controls versus APP/PS1, *p* = 1.38 × 10^−6^ for controls versus rTg4510, and *p* = 1.01 × 10^−12^ for APP/PS1 versus rTg4510. ***E***, Fractions of hyperactive neurons. Controls: 2.91 ± 0.35%, APP/PS1: 19.11 ± 1.50%, rTg4510: 0.93 ± 0.35%; *F*_(2,18)_ = 176.2, *p* = 1.51 × 10^−12^. *Post hoc* multiple comparisons were *p* = 2.84 × 10^−11^ for controls versus APP/PS1, *p* = 1.64 × 10^−12^ for APP/PS1 versus rTg4510 and not significant, *p* = 0.1045, for controls versus rTg4510. ***F***, Fractions of silent neurons. Controls: 15.05 ± 1.87%, APP/PS1: 9.20 ± 2.36%, rTg4510: 53.48 ± 3.24%; *F*_(2,18)_ = 77.18, *p* = 1.48 × 10^−9^. *Post hoc* multiple comparisons were *p* = 2.02 × 10^−8^ for controls versus rTg4510 and *p* = 1.08 × 10^−8^ for APP/PS1 versus rTg4510 and not significant, *p* = 0.3972, for controls versus APP/PS1. Each solid circle represents an individual animal (controls, *n* = 7; APP/PS1, *n* = 5; rTg4510, *n* = 9), and all error bars reflect the mean ± SEM; the differences between genotypes were assessed by one-way ANOVA followed by Tukey’s multiple comparisons test, ****p < 0.0001. NS, not significant. ***G***, ***H***, Neuronal activity is reduced in P301S mice independently of presence of NFTs. Reproduced with permission from Marinković et al. (2019). ***G***, left, Representative *in vivo* recordings from WT vehicle and P301S tau-PFFs (tau preformed fibrils-injected) mice. AAV1 transduced neurons are labeled with mRuby2 (red) and GCaMP6s (green). NFTs are labeled with FSB (white). Images are made by averaging 450 time-series frames acquired *in vivo* at 410 Hz with two-photon lasers tuned to 940 nm for CGaMP6s/mRuby2 and to 750 nm for FSB. Scale bar: 50 mm. Right, Traces (blue) extracted from annotated regions of interest (black) during quiet and active (gray shade) behavioral states classified based on changes in whisking movement (gray trace in the bottom). Note that traces 2 and 4 in P301S tau-PFFs group are from NFT-bearing neurons. Black bars mark detected calcium transients. ***H***, Mean frequency of calcium transients during quiet and active states of all neurons detectable in three or more time points. WT vehicle (black), WT tau-PFFs (cyan), P301S vehicle (green), all P301S tau-PFFs: all neurons are denoted as magenta circles, with NFT-free as magenta squares and NFT-bearing as magenta triangles. Data points represent individual mice, *n* = 5–7 mice per group; black lines represent mean value ± SEM; ***p < 0.001, ****p < 0.0001, WT versus P301S (two-way ANOVA, genotype factor, not significant; Student’s *t* test).

Interestingly, in stark contrast to the data supporting the notion that tau promotes neuronal network hyperexcitability in AD, some recent studies demonstrate that tau may actually suppress it. A recent study employing *in vivo* two-photon Ca^2+^ imaging of large populations of neurons in layer 2/3 of the neocortex showed that while Aβ promotes neuronal network hyperactivity, tau in fact suppresses the activity (Fig. 4*A–F*; Busche et al., 2019). The study found neuronal hyperactivity in 6- to 12-month-old plaque-bearing APP/PS1 mice, and a strong reduction of cortical activity levels in age-matched rTg4510 transgenic mice that express aggregating human tau P301L (expression level of human tau 13-fold higher than that of endogenous tau) and display NFTs but no Aβ pathology (Busche et al., 2019). Interestingly, tau aggregation was not necessary for neuronal silencing as suppression of cortical activity was found in neurons devoid of NFTs (Busche et al., 2019). Furthermore, marked reduction of neuronal activity was found in neurons of rTg21221 mice that overproduce non-aggregating WT human tau at comparable levels to rTg4510 mice but lack NFTs (Busche et al., 2019). These data suggested that impairment of neurons could occur with tau overexpression independent of tau aggregation and NFTs formation. This was further validated by reduction in cortical activity in young age rTg4510 mice (Busche et al., 2019). Soluble, non-aggregated tau was found to be sufficient for neuronal silencing, and NFTs were not required (Busche et al., 2019). Interestingly, on evaluation of Aβ and tau together, neuronal hyperactivity was not only completely abolished in the crossed APP/PS1-rTg4510 and APP/PS1-rTg21221 mice, but there was also a strong reduction in cortical activity levels, both in old and young mice (Busche et al., 2019). These data suggested that tau blocks Aβ-dependent hyperactivity, leading to silencing of circuits when both Aβ and tau are present together in the cortex. While these findings are contradictory to other data showing neuronal network hyperactivity in both young and aged 3xTg-AD mice which harbor both Aβ and tau along with PS1 (Davis et al., 2014; Kazim et al., 2017), they are supported by another study in young 3xTg-AD mice which reported a decrease in neuronal activity in these mice (Mondragón-Rodríguez et al., 2018), this will be discussed in more detail later in the present review (see below, Phosphorylation of tau reduces hippocampal excitability). Remarkably, while suppressing tau transgene expression resulted in reversal of suppression of neural activity in tau mice, it was less effective in rescuing neuronal network impairments in crossed mice containing both Aβ and tau (Busche et al., 2019), suggesting a complex interaction of Aβ and tau in neural activity.

Another recent study has reported similar results, i.e., reduced activity of neuronal circuits in a P301S mouse model of tauopathies (Fig. 4*G*,*H*; Marinković et al., 2019). By using chronic *in vivo* two-photon calcium imaging in awake mice, a strong reduction of calcium transient frequency in layer 2/3 cortical neurons of P301S mice, independent of NFTs presence, was found (Marinković et al., 2019). Interestingly, reduced neuronal activity in P301S mice did not change with time and pathology progression (Marinković et al., 2019). Thus, it was concluded that it is soluble, mutated tau protein species, not NFTs, that suppress neuronal activity (Busche et al., 2019; Marinković et al., 2019).

A previous study employing *in vivo* intracellular and extracellular electrophysiological recordings found that pathologic tau reduced the neocortical activity in rTg4510 mice before significant neurodegeneration and at an age where not all neurons in these mice express pathologic tau (Menkes-Caspi et al., 2015). The changes induced by pathologic tau included slower neuronal oscillations and reduced firing rates, and reduction in reliability of synaptic transmission in the transgenic neocortex (Menkes-Caspi et al., 2015). While the study did not differentiate between the effect of soluble tau and NFTs in suppressing network excitability, it was suggested that pathologic tau may in fact affect neuronal activity at levels below those detectable with routine immunocytochemical and perfusion methods (Menkes-Caspi et al., 2015). It is interesting to note here that these data are in contrast to other studies employing *in vitro* intracellular recordings in the same mice mentioned before (Rocher et al., 2010; Crimins et al., 2011, 2012). These differences could potentially be because of the inherent differences between preparations and recording techniques (Menkes-Caspi et al., 2015); however, further investigation is needed to clarify this discrepancy (we will discuss this in detail in Phosphorylation of tau reduces hippocampal excitability; and Perspectives on the Similar versus Divergent Roles of Aβ and tau in Neuronal Network Hyperexcitability in AD: Which One Has a Dominant Effect, Aβ or tau?).

In congruence with the findings of Menkes-Caspi and colleagues (Menkes-Caspi et al., 2015), another *in vitro* electrophysiological study reported tau-induced suppression of neuronal activity (Angulo et al., 2017). The study evaluated neuronal activity in mutant htau mice, mutant hAPP mice, and combined mutant htau and hAPP mice in the EC, one of the first regions in the brain to be affected by the AD pathology (mainly the tau pathology; Angulo et al., 2017). It was found that mutant EC-hAPP mice exhibited a significant increase in the duration of spontaneous extracellular field potentials (sEFPs) in EC (Angulo et al., 2017). Interestingly, pyramidal neurons of the subiculum in EC-hAPP mice, which are monosynaptically excited by EC layer III neurons, showed mEPSCs with reduced amplitude, suggesting that the increased excitation observed in EC induced a compensatory negative feedback in subicular projection neurons, a process known as synaptic homeostasis, explained by EC interneuron pruning based on computational modeling (Angulo et al., 2017). The physiological changes produced in EC by the expression of mutant tau protein (P301L) manifested as resistance to GABA_A_ receptor antagonist-induced hypersynchrony (Angulo et al., 2017). However, the human tau mutation, by itself, did not produce any significant spontaneous activity changes in EC-hippocampus circuits. Remarkably, mice exhibiting both Aβ and tau pathologies displayed an intermediate and subtler phenotype, which was predominantly driven by tau pathology. These data suggested divergent roles of Aβ and tau in neuronal excitability with Aβ promoting hyperexcitability and tau suppressing excitability and tau exerting a dominant effect in the presence of both pathologies. These data are in congruence with a recent *in vivo* study discussed earlier (Busche et al., 2019).

#### Phosphorylation of tau reduces hippocampal excitability

Phosphorylation of tau has been considered the most critical posttranslational modification in taupathies and neurodegenerative diseases (Busche and Hyman, 2020). tau protein contains 85 potential tyrosine (Y), threonine (T), and serine (S) phosphorylation sites. A comprehensive analysis of phosphorylation sites of tau protein has revealed >40 phosphorylation sites in AD (Morishima-Kawashima et al., 1995; Hanger et al., 2007). Accumulating evidence indicate that different phosphorylation sites result in changes in synaptic function, axonal initial segment, which ultimately precipitates to abnormal neuronal excitability and network dysfunction (Ittner et al., 2016; Mondragón-Rodríguez et al., 2018b; Hill et al., 2019). A study employing patch-clamp electrophysiology of hippocampal CA1 neurons in two tau pathology mouse models, the rTg4510 strain and a second model, pR5, that also expresses P301L mutant tau, although at much lower levels, showed that hyperphosphorylated tau before neurodegeneration induced a more depolarized threshold for action potential initiation and reduced firing in hippocampal CA1 neurons, an effect that was rescued by the suppression of transgenic tau (Hatch et al., 2017). The authors found that this reduction in neuronal excitability resulted from the relocation of the AIS down the axon in a tau phosphorylation-dependent manner, which was microtubule dependent. Interestingly, the authors found that the shift of AIS is correlated with phosphorylation of tau at pThr231/pSer235 and pSer262/pSer356, but not pSer396/pSer404, indicating that the sites of phosphorylation is critical for mediating reduction of hyperexcitability (Hatch et al., 2017).

A more recent study by Lennart and his colleagues demonstrated, by using P301L pR5 mouse model, that hyperphosphorylation of tau resulted in increase in stubby spines and filopodia, reduction of total dendritic length of hippocampal pyramidal neurons. The authors also found that the neuronal atrophy resulted in a significant reduction of LTP in CA1, depolarized threshold for action potential initiation, and an increased current of inward rectifying potassium channels (Müller-Thomsen et al., 2020). As results, hyperphosphorylation of tau lead to decreased excitability of CA1 neurons.

While these data are in contrast to other *in vitro* studies reporting hyperexcitability in rTg4510 mice (Rocher et al., 2010; Crimins et al., 2011, 2012), it must be noted that those previous studies analyzed excitability in cortical pyramidal neurons as compared with this study which evaluated CA1 hippocampal neurons. These observations also raise the possibility that neuronal dysfunction resulting from tau hyperphosphorylation may occur in a brain region-specific manner. Nonetheless, as mentioned before, recent *in vivo* studies have also found reduced excitability in the neocortex of rTg4510 mice (Menkes-Caspi et al., 2015; Busche et al., 2019).

Previously, also in rTg4510 mice, it was found that tau was aberrantly targeted to dendritic spines by the P301L mutation, before overt neurodegeneration and synaptic loss (Hoover et al., 2010). It was reported that phosphorylation controlled tau mislocalization to dendritic spines, and once mislocalized to spines, tau suppressed excitatory synaptic transmission and caused loss of surface AMPA receptors in spines (Hoover et al., 2010). These findings were reported both in rTg4510 mice cultured cortical neurons and in rat hippocampal neurons with transfected htau-P301L mutation (Hoover et al., 2010).

Remarkably, a protective role of site-specific phosphorylation of tau against Aβ excitotoxicity was recently reported (Ittner et al., 2016), challenging the dogma that tau phosphorylation only mediates toxic processes in AD. p38 mitogen-activated protein kinase (p38MAPK) is known to phosphorylate tau (Goedert et al., 1997; Feijoo et al., 2005; Cuenda and Rousseau, 2007). Although p38MAPK was reported to contribute to NMDAR-mediated toxicity (Mucke and Selkoe, 2012), and its inhibition improved Aβ-induced LTP deficits (Wang et al., 2004), paradoxically, hyperexcitability in APP transgenic mice increased with inhibition of p38MAPK (Ittner et al., 2014). Among different p38MAKs, p38MAPKγ was found to localize to dendritic spines and postsynaptic densities (PSDs) of neurons (Ittner et al., 2016). Depletion of postsynaptic p38MAPKγ exacerbated neuronal network hyperexcitability in APP23 transgenic mice (Ittner et al., 2016). Furthermore, it was reported that p38MAPKγ-mediated phosphorylation of tau at threonine 205 (Thr205) disrupted NR/PSD-95/tau/Fyn complexes, and reduced Aβ-dependent neuronal network hyperexcitability (Ittner et al., 2016). These data (Ittner et al., 2016), along with earlier data from the same group (Ittner et al., 2010), suggest that while tau may promote neuronal network hyperexcitability, the phosphorylation of tau in fact suppresses excitability and protects against excitotoxicity.

Another recent study also showed reduction in hippocampal excitability by phosphorylation of tau protein (Mondragón-Rodríguez et al., 2018b). The study found that pyramidal neurons in the hippocampus from young 3xTg-AD mice (one-month-old, without any cognitive deficit) accumulated hyperphosphorylated tau at the microtubule domain region (MDr) and exhibited reduced neuronal network excitability and seizure susceptibility. Further analysis showed that phosphorylation site at Ser396 was responsible for changes in network excitability in these mice (Mondragón-Rodríguez et al., 2018). Previously, the same group reported that tau phosphorylation at MDr sites could serve as a regulatory mechanism to prevent overexcitation (Mondragón-Rodríguez et al., 2012). Interestingly, the study also found alterations (increase) in network oscillatory activity at θ band frequency in young 3xTg-AD mice (Mondragón-Rodríguez et al., 2018). Previously, changes in hippocampal θ activity were correlated with the cognitive impairment observed during neurodegeneration (Cayzac et al., 2015). Specifically, slowing of hippocampal activity has been correlated with cognitive decline in early onset AD (Engels et al., 2016). It was postulated that the increase in θ activity observed at a young age in 3xTg-AD mice before any cognitive deficit and neurodegeneration could be an early compensatory response, and may contribute to early network dysfunction in these mice and AD (Mondragón-Rodríguez et al., 2018). Nonetheless, as 3xTg-AD mice also harbor APP and PS1 mutations in addition to a tau mutation, it is difficult to ascertain whether this increase in θ oscillations was a result of tau and its phosphorylation or APP/Aβ overexpression. In fact, a recent study showed early weakening of θ oscillations and drastic impairments in θ–γ oscillations phase-amplitude cross frequency coupling induced by tau seedings in a P301L human mutant tau mouse model (Ahnaou et al., 2017). Interestingly, the study failed to find any epileptiform activity or network hyperexcitability in these mice with tau aggregates induced by seeding, and argued toward a causal relationship between the early disruption in functional networks (but not neuronal network hyperexcitability) and tau aggregation (Ahnaou et al., 2017). Along similar lines, two recent studies showed that inducing γ oscillations via sensory stimulation (γ entrainment using sensory stimulus or GENUS) not only benefited cognition but also ameliorated Aβ and tau pathologies in 5XFAD and P301S mouse models of AD, respectively (Adaikkan et al., 2019; Martorell et al., 2019).

## Perspectives on the Similar versus Divergent Roles of Aβ and tau in Neuronal Network Hyperexcitability in AD: Which One Has a Dominant Effect, Aβ or tau?

The presence of neuronal network hyperexcitability in human MCI/AD patients and animal models of the disease is well documented (Palop et al., 2007; Crimins et al., 2011, 2012; Bakker et al., 2012, 2015; Vossel et al., 2013, 2016, 2017; Davis et al., 2014; Bezzina et al., 2015; Kazim et al., 2017). While routine scalp EEG recordings cannot always accurately detect epileptiform activity originating from the mesiotemporal region (including the hippocampal formation; Clemens et al., 2003; Nilsson et al., 2009) and carries low yield of identifying network hyperexcitability in AD (Horváth et al., 2016), neuronal hyperactivity, silent seizures, and spikes have been reported in the MCI patients’ hippocampus using techniques with better detection properties such as fMRI (Bakker et al., 2012, 2015) and foramen ovale electrode (Lam et al., 2017). As discussed in detail in this review, while the causal relationship between Aβ and network hyperexcitability remains to be established in human AD patients, the role of Aβ (even before the formation of plaques, i.e., intraneuronal hAPP/Aβ and soluble Aβ) in promoting neuronal network hyperexcitability has been consistently reported in animal model studies (Palop et al., 2007; Busche et al., 2008, 2012; Minkeviciene et al., 2009; Palop and Mucke, 2010a,b; Sanchez et al., 2012; Verret et al., 2012; Born et al., 2014; Bezzina et al., 2015; Kazim et al., 2017). However, the role of tau in network excitability in AD remains yet to be precisely delineated as different animal model studies reported divergent effects (Roberson et al., 2007; Crimins et al., 2011, 2012; Roberson et al., 2011; García-Cabrero et al., 2013; Menkes-Caspi et al., 2015; Angulo et al., 2017; Mondragón-Rodríguez et al., 2018b; Busche et al., 2019; Van Erum et al., 2020). Nonetheless, recent human studies have reported a correlation between temporal lobe tau accumulation, CSF tau levels and hippocampal hyperactivity (Berron et al., 2019; Huijbers et al., 2019), suggesting a proepileptic effect of tau. Importantly, as in human AD brain, the Aβ and tau pathologies co-exist and potentially both contribute to neurodegeneration, a highly relevant question that remains to be answered is how the two histopathological hallmarks interact to affect the neuronal network activity in AD brains, and which one has a dominant effect, Aβ or tau?

**Figure 5.**
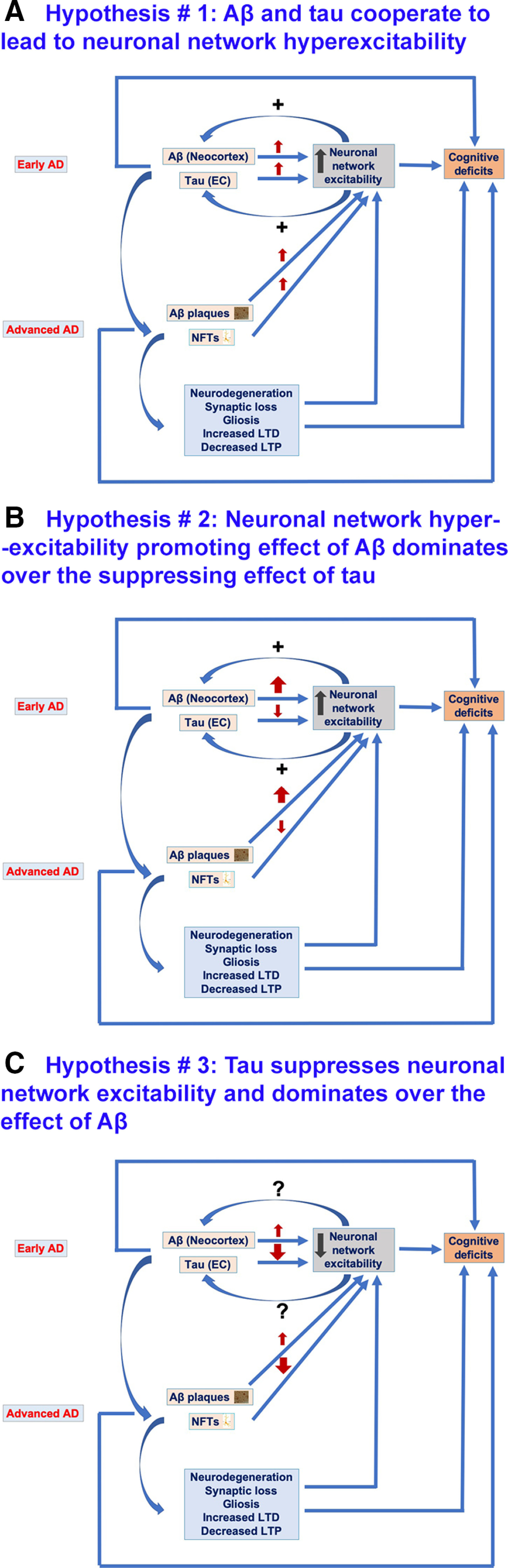
Hypotheses regarding the possible effects of Aβ and tau on neuronal network excitability in AD. ***A***, Hypothesis #1, Aβ and tau cooperate to lead to neuronal network hyperexcitability in AD. At early stages of AD, Aβ is more abundant in the neocortex whereas tau is localized to EC. Both Aβ and tau at early AD stages promote neuronal network hyperexcitability which not only contributes to cognitive impairments but also reciprocally increases Aβ deposition and tau release and spread to other cortical areas across connected neuroanatomical circuitry. Also, at advanced AD stages, both Aβ and tau promote neuronal network hyperexcitability, thus leading to cognitive deficit. Furthermore, Aβ-induced and tau-induced neuronal and synaptic loss, gliosis, and impaired synaptic plasticity (decreased LTP and increased LTD) contribute to neuronal network hyperexcitability and to cognitive deficits, effects also at play in scenarios illustrated in ***B***, ***C***. ***B***, Hypothesis #2, Aβ enhances neuronal network hyperexcitability whereas tau suppresses excitability; the overall phenotype is hyperexcitability as Aβ effect dominates over tau effect. Aβ at early AD stages promotes neuronal network hyperexcitability which not only contributes to cognitive impairments but also increases Aβ deposition and tau release and spread to other cortical areas across connected neuroanatomical circuitry. However, tau at early AD stages suppresses neuronal activity, thus leading to silencing of neuronal networks which could also contribute to AD-related network dysfunction and cognitive deficit. Also, at advanced AD stages, Aβ enhances and tau suppresses neuronal network excitability, both leading to cognitive deficits. This could also be the case in AD patients with higher Aβ deposits than NFTs in their brains. ***C***, Hypothesis #3, tau suppresses neuronal network excitability, whereas Aβ enhances it; the overall phenotype is suppressed excitability as tau suppressive effect dominates over Aβ enhancing effect. Tau both at early and advanced AD stages suppresses neuronal excitability thus leading to silencing of neuronal networks contributing to AD cognitive deficits. Contrarily, Aβ both at early and advanced AD stages promotes neuronal network hyperexcitability however this is dominated by tau suppressive effect. However, this hypothesis cannot explain the tau spread from EC to other cortical areas as increased neuronal activity has been identified to promote propagation of tau. Nonetheless, there could be other mediators of tau spread besides neuronal network hyperexcitability.

With subclinical epileptiform activity and network hyperactivity demonstrated in human MCI/AD patients (Bakker et al., 2012, 2015; Vossel et al., 2013, 2016; Lam et al., 2017), it seems plausible that either Aβ and tau cooperate to lead to neuronal network hyperexcitability (Fig. 5*A*, hypothesis #1) or the neuronal network hyperexcitability promoting effect of Aβ dominates over the suppressing effect of tau (Fig. 5*B*, hypothesis #2; if tau suppresses neuronal network activity as suggested by recent animal model studies; Hatch et al., 2017; Mondragón-Rodríguez et al., 2018b; Busche et al., 2019; Marinković et al., 2019). In support of both the first and second hypotheses, few studies in AD transgenic mice harboring both Aβ and tau (3xTg-AD) have reported hyperexcitability in these mice both at early and late stages of the pathology (Davis et al., 2014; Kazim et al., 2017). In agreement with the first hypothesis, tau reduction in hAPP mice has been reported to decrease neuronal network excitability (Roberson et al., 2007; Ittner et al., 2010; Roberson et al., 2011).

Alternatively, another line of evidence suggests that tau may be the dominant protein over Aβ, and that it causes overall suppression of neuronal hyperexcitability. This was demonstrated by Busche and colleagues, who used *in vivo* calcium imaging of cortical neurons from APP/PS1-rTg4510 and APP/PS1-rTg21221 mice (Busche et al., 2019).

Neuropathological autopsy studies and more recently neuroimaging (PET scan) studies have shown plaques and NFTs deposition in AD brains differ both spatially and temporally from each other (Arnold et al., 1991; Braak and Braak, 1991, 1995, 1996; Schöll et al., 2016). In AD brains, the Aβ plaques first form in the neocortex and other cortical areas and then spread inward to deeper brain regions, whereas NFTs first form in the EC within the hippocampal formation and limbic areas, and from there they spread outward to the neocortex and other cortical areas (Arnold et al., 1991; Braak and Braak, 1991, 1995, 1996; Schöll et al., 2016). Neuronal activity was reported to enhance tau release and propagation (Pooler et al., 2013; Wu et al., 2016). Accumulating evidence supports the notion that the spread of tau from EC and limbic regions to other neocortical areas coincides with the appearance of cognitive impairment in AD (Wang et al., 2016; Bejanin et al., 2017; Pontecorvo et al., 2017; Jagust, 2018). Despite being a neuropathological hallmark of AD, Aβ correlates weakly with neurodegeneration; rather, it is tau that is associated with brain atrophy and hypometabolism, which, in turn, are related to cognition (Nelson et al., 2012; Bejanin et al., 2017; Jagust, 2018). It thus remains possible that in human AD brains, Aβ-induced neuronal network hyperexcitability enhances propagation of tau from EC to Aβ-bearing neocortex which leads to emergence of cognitive deficit.

While several studies over the past two decades have demonstrated that the interaction between Aβ and tau leads to increased pathology (Götz et al., 2001; Lewis et al., 2001; Hurtado et al., 2010; Wang et al., 2016; Bennett et al., 2017; Pontecorvo et al., 2017; Jacobs et al., 2018), the physiological consequences of this interaction on neuronal network excitability in AD is a matter of debate. As discussed before, there is strong experimental evidence for neuronal network hyperexcitability enhancing the effects of both Aβ and tau. In this context, how can we explain the neuronal activity suppressing effect of tau demonstrated in recent animal model studies? As discussed by Busche et al. (2019), studies have also showed a progressive reduction in whole-brain activity in AD patients (Silverman et al., 2001; Alexander et al., 2002; Greicius et al., 2004) and regional cerebral blood flow (Bradley et al., 2002) as well as an EEG slowing (Jelic et al., 1997), all of which could be explained by a dominant neuronal activity suppressive effect of tau. Furthermore, the dominant role of tau along with the well-reported data that tau correlates with cognitive decline better than Aβ (Nelson et al., 2012) could also explain the failure of a number of Aβ-based AD clinical trials. Nonetheless, as mentioned before, subclinical epileptiform activity and neuronal network hyperactivity have been well documented in human MCI/AD patients (Bakker et al., 2012, 2015; Vossel et al., 2013, 2016; Lam et al., 2017). As neuronal network hyperexcitability is more clearly linked to Aβ (Palop and Mucke, 2010a,b, 2016; Zott et al., 2018), it is possible that epileptiform activity (as detected by EEG) is more prominent in AD patients who have comparatively higher Aβ than tau levels, or are at early stages of the disease when Aβ is present in the cortex but tau is limited to EC and limbic areas. Also, in this context, it must be mentioned here that the predominant subconvulsive epileptiform activity phenotype observed in mice carrying hAPP mutation(s), could primarily be because of transgenic mutant APP, as suggested by some studies (Born et al., 2014; Kazim et al., 2017).

It is prudent to mention here that there a few other factors that need to be taken into consideration when evaluating the conflicting data from animal models regarding the roles of Aβ and tau in neuronal network hyperexcitability in AD. First, it is important to consider at what level of the brain organization hierarchy (neuronal, synaptic, circuit, or network) the evaluation was performed. The single-neuron data may differ from a neuronal population circuit or network data. A good example of differential effects at different levels of organization is synaptic depression versus aberrant excitatory network activity induced by Aβ (Palop and Mucke, 2010a,b). In AD experimental models, pathogenic Aβ reduces glutamatergic transmission and enhances LTD at the synaptic level (Hsia et al., 1999; Kamenetz et al., 2003; Hsieh et al., 2006; Palop et al., 2007) whereas at the network level, Aβ causes dysrhythmias, including neuronal synchronization, epileptiform activity, and seizures (Palop et al., 2007; Busche et al., 2008). Both synaptic depression and aberrant network synchronization probably interfere with activity-dependent synaptic regulation, essential for learning and memory (Palop and Mucke, 2010a,b). Similarly, tau causes synaptic loss (Forner et al., 2017) and impairs LTP (Fá et al., 2016; Puzzo et al., 2017) at the synapse level which would lead to slowed neuronal activity, whereas at the network level both increase and decrease of network activity have been reported in tau mouse models of AD (as discussed before in this review). Second, the extent and exact sites of tau phosphorylation may have to be taken into account to determine whether its overall effect opposes or contributes to the neuronal network hyperexcitability. A third important factor to consider when interpreting neuronal network hyperexcitability in AD studies is to take into account the role of other pathophysiological features of AD such as neuronal loss, gliosis, and E/I imbalance in enhancing hyperexcitability (Miranda and Brucki, 2014; Busche and Konnerth, 2016; Zott et al., 2018; Vico Varela et al., 2019).

## Future Directions and Concluding Remarks

Neuronal network hyperexcitability has been identified as an important component of AD pathophysiology and potentially contributes to cognitive deficit in AD. Evidence for Aβ promoting neuronal network hyperexcitability in AD, demonstrated by both *in vitro* and *in vivo* models, strongly suggest network hyperexcitability role of Aβ. However, the role tau, either by itself or in combination with Aβ, on network excitability in AD need to be more carefully elucidated. To this end, there has been a significant effort to create humanized animal models of tau and Aβ, which have become invaluable tools to recapitulate tau pathology in AD. Many recent studies use these models, where tau extracted from brains of AD patients is injected into the brains of humanized tau mice. Such studies, combined with *in vivo* and *in vitro* electrophysiology, should provide insight into potential role of tau in network excitability. Furthermore, we discussed that an incongruity among studies addressing the role of tau in enhancement versus suppression of neuronal network excitability come from brain region and cell type specificity; studies for network enhancement use cortical neurons, while studies for network suppression come from hippocampal neurons (Ittner et al., 2010, 2016; Crimins et al., 2012, 2013; Kopeikina et al., 2013; Mondragón-Rodríguez et al., 2018b). It is entirely possible that the impact of tau on network excitability is brain region dependent; *in vivo* experiments with multiple tau injections at different brain regions, paired with *in vivo* calcium imaging and electrophysiology may provide insight into region-specific effect of tau on network excitability. This area of investigation may provide the necessary knowledge to develop more effective and refined strategies for prevention, diagnosis, and management of AD and related dementias.

## Synthesis

Reviewing Editor: Karl Herrup, University of Pittsburgh School of Medicine Decisions are customarily a result of the Reviewing Editor and the peer reviewers coming together and discussing their recommendations until a consensus is reached. When revisions are invited, a fact-based synthesis statement explaining their decision and outlining what is needed to prepare a revision will be listed below. The following reviewer(s) agreed to reveal their identity: NONE.

This review offers a comprehensive examination of the recent literature on the impact of APP/Abeta and tau on neuronal and network hyperexcitability in mouse models of Alzheimer’s disease. The authors consider both Aβ and tau models. This is a timely and important topic. The reviewers noted that several other similar reviews have appeared recently, but both felt that this submission would add value to the existing literature because of its detail and breadth of coverage as well as its efforts to tie findings from animal models to human disease pathophysiology. The tables were also singled out for praise being rated as quite useful. Despite these strengths there are concerns that, if addressed, would strengthen the manuscript.

Speaking to the growing body of literature in this area, it was deemed unfortunate that no literature from 2020 is discussed. Several recent preprints and published articles are relevant and should be included in the discussion (e.g., Jun et al, Neuron 2020, as well as others). The section on Abeta was fairly straightforward and well received. There were cogent suggestions, however, for the tau section. tau is a protein with multiple actions, and it can be manipulated in different directions - reduction of endogenous gene expression, overexpression of human/mutant tau, etc. This divergence of function offers one reason why the current literature often seems scattered: many different facets of tau cell biology are lumped together: Paying closer attention to the details of how tau is altered in the various studies would be an important contribution for the authors to consider.

The section on tau phosphorylation can be seen as falling into this trap by treating tau phosphorylation as something monolithic, lumping various phosphorylation sites together under the same umbrella. While the details are not ironed it nonetheless seems clear that some tau phosphorylation sites have different effects than others. While there is an almost unavoidable impulse to lump/simplify, in this case oversimplification of a problem of this complexity can lead to some of the apparent inconsistencies.

There was concern that the three hypotheses illustrated in Figure 5 are confusing. One major reason appears to be that Hypothesis #3 addresses the issues of a hypoexcitable brain. This is fundamentally different than the issues surrounding a hyperexcitable brain that are dealt with in Hypotheses #1 and #2. One suggestion would be to start with the phenomenon you are hoping to explain mechanistically and develop a hypothesis from there rather than having hypotheses that explain a smorgasbord of loosely related things. This is especially true in this case as the review builds a strong case for hyperexcitability in AD.

Finally, there was sentiment expressed for including a section on future directions to guide the reader on what to look for in the future.
